# Health behaviour and wellbeing trends among Australian adults before and during the COVID-19 pandemic (2017–2022): An interrupted time-series analysis

**DOI:** 10.1016/j.pmedr.2024.102861

**Published:** 2024-08-21

**Authors:** Sarah Marshall, Bronwyn McGill, Christian Young, Philip Clare, Sarah Neill, Margaret Thomas, Adrian Bauman

**Affiliations:** a Prevention Research Collaboration, Sydney School of Public Health, Faculty of Medicine and Health, the University of Sydney, Camperdown, NSW, Australia; b Charles Perkins Centre, the University of Sydney, Camperdown, NSW, Australia; c NSW Biostatistics Training Program, NSW Ministry of Health, Australia; d National Drug and Alcohol Research Centre, UNSW Sydney, NSW, Australia; e Centre for Population Health, NSW Ministry of Health, Australia

**Keywords:** Prevalence, Population health, Surveillance, Health risk behaviors

## Abstract

**Objective:**

To explore population-level trends in health behaviours and wellbeing indicators from before (2017–2019) to during (2020–2022) the COVID-19 pandemic in Australia.

**Methods:**

Using cross-sectional data from New South Wales Adult Population Health Surveys (2017–2022, n = 73,680 responses), we calculated weighted prevalence estimates and interrupted time-series logit models to investigate trends in health behavioural risk factors (vegetable and fruit intake, physical activity, alcohol consumption, smoking, e-cigarette use), Body Mass Index (BMI) (overweight and obesity), and wellbeing indicators (psychological distress, self-rated health) among adults aged ≥ 16 years before and during the pandemic.

**Results:**

From 2017 to 2022, the behavioural risk factors and BMI trends were mostly unchanged. Similarly, wellbeing indicator trends showed only minor variations according to age. The interrupted time-series models found marked changes from before to during the COVID-19 pandemic for e-cigarette use and self-rated health. E-cigarette use showed an overall increasing trend, with significant increases from 2017 to 2022 (OR 8.25, 95 %CI 6.10–11.16). Poor self-rated health showed a stable trend before COVID-19, but decreased in 2020 (OR 0.68, 95 %CI 0.58–0.80) and 2021 (OR 0.70, 95 %CI 0.60–0.81), returning to pre-COVID levels in 2022 (OR 1.23, 95 %CI 1.07–1.41). During the pandemic (2020–2022), there were few statistically significant observed changes in prevalence trends according to SES indicators.

**Conclusion:**

Among Australian adults, relatively small population-level impacts of the COVID-19 pandemic on health behaviours and wellbeing trends were observed. Continued surveillance and sub-group analyses are essential for investigating potential time-lagged effects and regional or sociodemographic differences in health behaviours and wellbeing.

## Highlights

• We explored health behaviour and wellbeing trends in NSW, Australia, 2017–2022.
• Our analysis found limited population-level impacts of the COVID-19 pandemic.
• Continued monitoring should investigate potential delayed or ongoing effects.
• Socioeconomic disparities must remain a priority for preventive health measures.

## 1 Introduction

Modifiable behaviours, including being physically active, healthy eating, not smoking and consuming alcohol within recommended levels can promote health and reduce the risk of morbidity and death related to non-communicable diseases (GBD, 2017, Zhang et al., 2021). Globally, non-communicable diseases, such as chronic heart disease and diabetes, account for 74 % of all deaths (World Health Organization, 2022). Evidence shows that the population distribution of non-communicable diseases and related mortality is skewed toward those experiencing poverty or social disadvantage regardless of country (World Health Organization, 2010, Lago-Peñas et al., 2021). Mental health is an integral facet of health and wellbeing, impacting individuals’ productivity and potential. Positive mental health and psychological wellbeing are associated with increased physical activity and healthy eating behaviours (Heinsch et al., 2022). At a population level, understanding health and wellbeing trends can inform equitable health resource allocation and targeted public health action.

On 11 March 2020 the World Health Organization declared the COVID-19 outbreak a pandemic (World Health Organization, 2005). The pandemic had a societal-level impact on physical and mental health. The disease itself and the associated restrictions to reduce COVID-19 transmission continue to have consequences globally (Pak et al., 2020). Research into the impact of COVID-19 on population health behaviours and wellbeing is still emerging.

Many studies of lifestyle risk behaviours among adults during the COVID-19 pandemic tend to show negative trends, including increased sedentary time (Runacres et al., 2021, Stockwell et al., 2021 Feb 1), decreased physical activity (Stockwell et al., 2021 Feb 1, Amo et al., 2022, de Boer et al., 2021, Ammar et al., 2020, Wunsch et al., 2022 Feb 16), poorer dietary habits (Mekanna et al., 2023 Jun 9, Bakaloudi et al., 2022, Bennett et al., 2021), and increased alcohol consumption (Bakaloudi et al., 2022, Roberts et al., 2021 Dec 1). In contrast, one systematic review found reduced cigarette and e-cigarette use (Almeda and Gómez-Gómez, 2022). Other systematic reviews reported adverse psychological outcomes during the COVID-19 pandemic, including stress, anxiety and depression (Arora et al., 2022, Salari et al., 2020, Brooks et al., 2020). Importantly, these population-level trends are potentially country-specific and dependent on COVID-19 case prevalence, the type and duration of COVID-19 restrictions, and local cultural and societal norms.

The research literature regarding the impact of COVID-19 on health behaviours and wellbeing in Australia is limited. Reports indicate mixed implications for health behaviours and psychological wellbeing, and limited population trend data is available (Australian Institute of Health and Welfare, 2021). Notably, Australia’s experience of COVID-19 in 2020–2022 differed from many other countries. National and state/territory governments enforced widespread public health and social measures including stay-at-home orders (lockdowns), physical distancing, travel bans and restricted movement, cancellation of large gatherings, and only essential services were permitted to operate. As a result, in 2020, Australia maintained relatively low COVID-19 case numbers (approximately 28,500) and low deaths (approximately 900) (Australian Institute of Health and Welfare, 2022). In 2021, cases and deaths increased, particularly in the most populous states of Victoria and New South Wales, and lockdowns were enacted. In early 2021, COVID-19 vaccination commenced, and by June 2022, vaccination rates were reaching the population, and most prevention measures had eased, consistent with the control of other respiratory viruses.

Given the differences in COVID-19 cases and preventive measures across Australia, and when compared internationally, a state-level trend analysis can provide important context-relevant insights. This study explored population-level trends in health behavioural risk factors and wellbeing indicators from before (2017–2019) to during (2020–2022) the COVID-19 pandemic in New South Wales, Australia, and examined differences according to socioeconomic indicators. Our objective was to understand the effect of the pandemic on adult health and wellbeing trends, and to inform public health policy and practice for improving population health outcomes.

## 2 Methods

### 2.1 Data source and study population

This study used data from the annual New South Wales (NSW) Adult Population Health Surveys, before (2017–2019) and during (2020–2022) the COVID-19 pandemic. The survey is conducted via telephone interview with a sample of adults (aged 16 years and over) residing in NSW (NSW Health, 2023). NSW is the most populated state in Australia, with most residents living in Sydney. Recruitment was state-wide via list-assisted random digit dialling using a dual landline- and mobile-phone sampling frame before 2021 (Barr et al., 2012) and a completely mobile-phone based sampling frame in 2021 and 2022. The survey questionnaires, available online (NSW Health, 2023), include questions about health behaviours, health status, and sociodemographic characteristics. Data collection occurs annually from February to December; however, in 2020 during the first national lockdown (late March to early May 2020) no data were collected, after which data collection resumed as usual. The NSW Adult Population Health Surveys were approved by the NSW Population and Health Services Research Ethics Committee. All survey participants provided informed verbal consent.

### 2.2 Measures

The variables used for this study comprise sociodemographic characteristics, health behaviours, Body Mass Index (BMI), wellbeing indicators and socioeconomic indicators (listed in Supplementary Table 1).

#### 2.2.1 Health behavioural risk factors, Body Mass Index, and wellbeing indicators

Health-related indicators were: insufficient vegetable and fruit intake, insufficient physical activity, excessive alcohol consumption, cigarette and e-cigarette use, Body Mass Index (BMI), psychological distress, and self-rated general health.

Vegetable and fruit intake was measured using serves consumed per day, with serve sizes aligned with the Australian Dietary Guidelines (National Health and Medical Research Council, 2013). Because fewer than 90 % of Australian adults meet the Australian Dietary Guidelines recommendation of at least five serves of vegetables per day (Australian Bureau of Statistics, 2022), we used < 3 serves per day as a pragmatic cut-off for insufficient intake. This cut-off aligns with government monitoring of vegetable intake and has been used by others (Centre for Epidemiology and Research, 2008, Ding et al., 2015, Cranney et al., 2022). Insufficient fruit intake was categorised as < 2 serves per day as per the Australian Dietary Guidelines for fruit intake. Physical activity was assessed using minutes of moderate-to-vigorous-intensity physical activity (MVPA) per week, including minutes of walking, and with vigorous physical activity weighted by two. We categorised < 150 min per week as insufficient physical activity, and also included categories of 150–300, or > 300 min per week, to align with the 2020 World Health Organisation’s recommendation of 150–300 min of moderate to vigorous aerobic activity per week for all adults (World Health Organization, 2020). Excessive alcohol consumption was categorised as > 10 standard drinks per week, where one standard drink equals 10 g of pure alcohol, which aligns with the Australian guidelines to reduce health risks for drinking alcohol (National Health and Medical Research Council, 2020). Tobacco cigarette smoking and e-cigarette use variables were dichotomised as yes/no, based on reported daily or occasional use.

BMI is an established international tool to categorise body weight, and the population is categorised as underweight (<18.5 kg/m^2^), healthy weight (18.5–24.9 kg/m^2^), overweight (25–29.9 kg/m^2^), and obese (30 + kg/m^2^). We considered the overweight and obese categories independently given the different health risks (GBD 2015 Obesity Collaborators et al., 2017).

Psychological distress was measured using the validated Kessler Psychological Distress Scale (K10), a 10-item questionnaire about anxiety and depressive symptoms over the last four weeks. Each item was rated on a 5-point scale and the total score calculated (range 10–50). Total scores were dichotomised using established cut-offs (Australian Bureau of Statistics, 2012): low or moderate (<22) or high or very high (≥22) psychological distress. The K-10 is usually measured in alternate years, however, was included in the 2020 survey and removed again in 2022; therefore, K10 data from 2017, 2019, 2020, 2021 was available for this study. For self-rated general health, participants were asked to rate their general health during the past four weeks using a single question with a 6-point scale. Responses were dichotomised as excellent/very good/good/fair and poor/very poor.

#### 2.2.2 Socioeconomic status

We used three indicators for socioeconomic status (SES): area-level disadvantage, level of remoteness and educational attainment. Residential postcode was coded against the Socioeconomic Indexes for Areas (SEIFA) Index of Relative Socioeconomic Disadvantage (IRSD) (Australian Bureau of Statistics, 2016). This index summarises the economic and social conditions of people and households within a postcode area and measures relative disadvantage. The index scores were categorised as quintiles, from quintile 1 (least disadvantaged) to quintile 5 (most disadvantaged). Postcode was also coded against the Accessibility-Remoteness Index of Australia (ARIA+) (Australian Bureau of Statistics, 2021). This index provides an indicator of relative access to services, dichotomised as 1) major city and inner regional (good access), and 2) outer regional, remote, and very remote (less access). Educational attainment was determined by participants’ highest educational level and categorised as: High school grade 10 or less, High school grade 12/TAFE/Diploma, or University or greater.

#### 2.2.3 Covariates

All models controlled for the participants’ age, sex, language spoken at home, and country of birth. For the primary analysis, age was treated as a continuous variable. For supplementary analyses, age was categorised as 16–24, 25–64, and ≥ 65 years. Participants’ sex was categorised as male or female. From 2020, this question was adjusted to gender, and the option of ‘other’ was added, Due to a limited sample size for ‘other’, our analyses included only male and female categories. Language other than English spoken at home was dichotomised to yes/no. We categorised countries of birth according to the Human Development Index (very high/high/medium levels) (United Nations Development Programme, 2023).

### 2.3 Statistical analysis

Each year of Population Health Survey data is weighted by the NSW Ministry of Health to account for potential sampling biases and achieve population representativeness. We first described sample characteristics for the years 2017–2022 using unweighted data. All further statistical modelling included survey strata and weights.

Initially, we estimated the unadjusted prevalence for each health outcome by year (2017–2022). We also estimated prevalence stratified by each of SEIFA, ARIA, and participants’ highest level of education, to show unadjusted trends in each SES group. We then assessed the impact of SES by estimating the prevalence of each health outcome in each SES group, based on SEIFA, ARIA or participants’ highest level of education as predictors, controlling for age, sex, language spoken and Human Development Index, allowing prevalence to vary both over time and SES group.

To assess the impact of the COVID-19 years, 2020–2022, we conducted a series of interrupted time-series (ITS) models, regressing each health outcome separately by year, estimated using generalised linear models. We assessed outcomes with binary responses using logit models, and outcomes with more than two response categories using multinomial logit models. To account for the impact of the 2020–2022 years, we included dummy variables to model both the difference between the predicted prevalence of each health outcome in 2020 and that of the counterfactual model (i.e., if no effect was present), and the difference in slope in 2020–2022 compared to the previous years. All ITS models were adjusted for age, sex, language spoken, Human Development Index, SEIFA, ARIA and highest level of education.

Logit models make two main assumptions: that covariates are not highly correlated (‘multicollinearity’) and that covariates have a linear association in the log odds. We assessed multicollinearity using variance inflation factors (VIF) (Thompson et al., 2017). The highest VIF observed was 2.16, suggesting issues due to multicollinearity are unlikely (Supplementary Table 2). We tested the functional form of age using multivariable fractional polynomial models, a data-adaptive method of assessing non-linear relationships that results in parameters that are linear in the log odds (Royston and Altman, 1994).

Unadjusted results are reported as weighted prevalence (%). The results of the ITS analyses are reported as odds ratios (ORs). We used an alpha of 0.05 and reported 95 % confidence intervals (95 %CI) for all estimates. All analyses were conducted in R, version 4.0.3 (R Core Team, 2018), and Stata, version 11.2 (StataCorp., 2009).

#### 2.3.1 Missing data

Participants could choose not to answer individual questions; therefore, 39.2 % of cases contained some missing data (2.6 % of total information). Because missing data can introduce bias and reduce power, we conducted analyses using multiple imputation. We imputed the data using the R package ‘mice’ (van Buuren, 2011), including all health outcomes and sociodemographic covariates as well as accounting for the complex survey (strata and weights). We created 20 imputed data sets with 100 iterations each. Continuous outcomes were imputed using predictive mean matching, and random forests were used to impute categorical outcomes. We used Rubin’s rules to pool parameter estimates from each imputation.

## 3 Results

### 3.1 Description of sample

From 2017 to 2022, we analysed 73,680 survey responses completed by NSW adults aged 16 years or older (11,417 – 13,287 survey completions per year). Sociodemographic characteristics (unweighted and unadjusted) were relatively constant over the time 2017–2020, but in 2021–2022, the sample had a lower mean age, a higher proportion of males and a higher proportion with university education (Table 1). Also, in 2021 and 2022, compared to other years, there were more respondents physically active for ≥ 300 min per week and more e-cigarette users.

**Table 1:** Sociodemographic and health data for adult population survey respondents in New South Wales, Australia, 2017–2022 (unadjusted, unweighted).

**Variable**	**Categories**	**2017**	**2018**	**2019**	**2020**	**2021**	**2022**
Total respondents (n)		13,287	13,161	12,801	11,657	11,417	11,357

Age (mean (SD))		59 (18)	60 (18)	61 (18)	62 (18)	50 (17)	50 (17)

Age group (%)	16–24 years	6.6	6	5.3	5.2	7.2	7.1
	25–64 years	47.9	44.9	42.9	40.5	70.1	68.6
	65 + years	45.5	49.1	51.8	54.3	22.6	24.2

Sex (%)	Females	56.5	57.0	56.2	56.8	53.4	54.3
	Males	43.5	43.0	43.8	43.2	46.6	45.7

Educational attainment (%)	High school grade 10	27.0	25.9	24.3	23.3	12.5	13.1
	High school grade 12/TAFE/Diploma	42.3	43.0	45.0	44.0	45.2	44.5
	University or greater	29.2	29.9	29.3	30.8	41.8	41.6

Area-level disadvantage (SEIFA IRSD^a^) (%)	Quintile 1 (least disadvantaged)	13.3	13.7	14.2	17.2	17.6	16.9
	Quintiles 2–4	62.9	63.1	63.8	65.4	68.0	68.1
	Quintile 5 (most disadvantaged)	23.8	23.2	22.0	17.3	14.4	15.0

Level of remoteness (ARIA+^b^) (%)	Metro/inner regional	85.1	84.9	85.0	86.4	88.7	88.9
	Outer regional/remote/very remote	14.9	15.1	15.0	13.6	11.3	11.1

Language other than English spoken at home (%)	No	87.0	86.8	87.9	87.5	85.8	85.2
	Yes	12.9	13.1	11.9	12.3	14.0	14.6

Vegetable intake (%)	< 3 serves per day	60.2	62.2	61.6	62.2	65.5	68.4
	≥ 3 serves per day	37.1	35.4	34.5	34.3	32.8	29.9

Fruit intake (%)	< 2 serves per day	51.2	54.3	54.6	54.2	59.8	62.2
	≥ 2 serves per day	47.5	44.4	43.3	43.9	39.1	36.9

Physical activity level (MVPA) (%)	< 150 min per weeks	43.7	42.9	44.5	43.4	32.9	35.4
	150 to < 300 min per week	15.9	16.6	15.5	16.2	15.8	16.0
	≥ 300 min per week	40.2	40.3	39.8	40.1	51.1	48.4

Alcohol consumption (%)	≤ 10 standard drinks per week	84.2	84.0	83.4	83.8	82.2	83.4
	> 10 standard drinks per week	14.9	14.8	15.0	14.5	16.8	15.7

Current tobacco cigarette smoking (%)	No	88.2	88.7	88.3	89.7	86.4	86.8
	Yes	11.6	11.2	11.4	9.9	13.3	12.8

Current e-cigarette use (%)	No	99.0	98.8	98.6	98.7	95.7	94.8
	Yes	0.8	0.9	1.1	1.0	4.0	5.0

Body Mass Index (BMI) category (%)	Underweight	2.6	2.3	2.6	2.3	1.5	1.9
	Healthy weight	35.6	35.5	33.9	34.9	33.8	33.6
	Overweight	32.5	32.6	32.9	32.8	34.8	33.9
	Obese	24.6	24.8	25.5	26.0	27.9	28.7

Psychological distress (K10^c^ score) (%)	Low or moderate (<22)	84.4	NA	82.7	79.8	82.2	NA
	High or very high (≥22)	12.6	NA	14.2	12.7	16.7	NA

Self-rated general health (%)	Excellent/very good/good/fair	90.6	90.3	90.1	92.1	93.8	90.2
	Poor/very poor	8.9	9.4	9.5	7.6	6.1	9.6

Abbreviations: NA=not available; SD=Standard deviation.

^a^SEIFA IRSD=Socioeconomic Indexes for Areas, Index of Relative Socioeconomic Disadvantage.

^b^ARIA+ = Accessibility-Remoteness Index of Australia.

^c^K10 = 10-item Kessler Psychological Distress Scale.

### 3.2 Trends in risk factors and wellbeing indicators

**Fig. 1:**
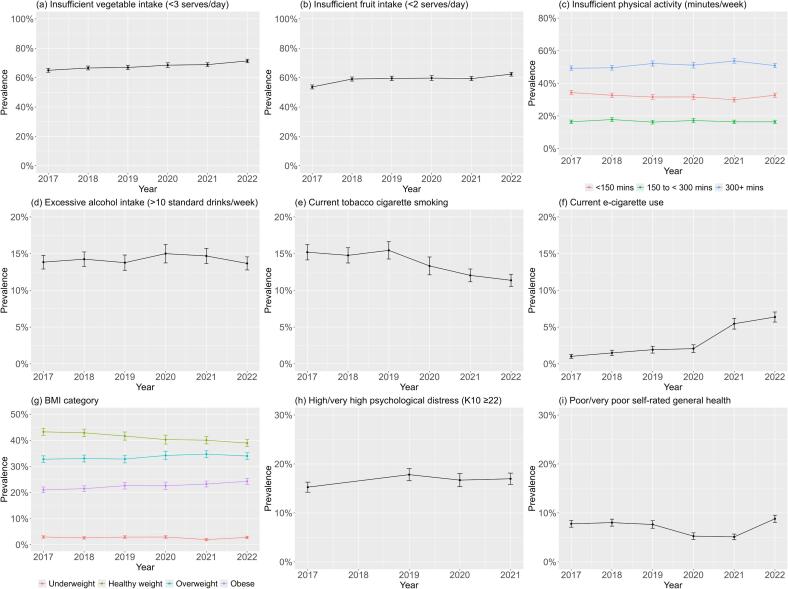
Weighted prevalence of health risk behaviours, BMI category, and wellbeing indicators among adults in New South Wales, Australia, 2017–2022.

The trend for excessive alcohol consumption was relatively stable. Tobacco cigarette smoking prevalence decreased from 15.2 % (95 %CI 14.2–16.3) in 2017 to 11.4 % (95 %CI 10.6–12.2) in 2022; however, e-cigarette use increased from 1.0 % (95 %CI 0.7–1.3) in 2017 to 6.4 % (95 %CI 5.7–7.1) in 2022, as shown in Fig. 1 and Supplementary Table 3.

There were increasing prevalences from 2017 to 2022 for the following: insufficient vegetable intake 65.0 % (95 %CI 63.7–66.3) to 71.4 % (95 %CI 70.1–72.6); insufficient fruit intake 53.7 % (95 %CI 52.3–55.0) to 62.4 % (95 %CI 61.0–63.7); overweight BMI category 32.8 % (95 %CI 31.5–34.1) to 34.0 % (95 %CI 32.7–35.3); obesity BMI category 21.1 % (95 %CI 20.0–22.1) to 24.3 % (95 %CI 23.2–25.4). Moderate-to-vigorous physical activity (MVPA) was relatively stable, with some increased prevalence in the 300 + mins/day category 49.3 % (95 %CI 47.9–50.7) in 2017, 53.7 % (95 %CI 52.3–55.2 %) in 2021, and back to 50.9 % (95 %CI 49.5–52.3) in 2022, and decreased prevalence in the < 150mins/day category. Supplementary analyses of physical activity with and without minutes of walking showed no notable difference in category trends (Supplementary Figure 1).

High or very high psychological distress (K10 score ≥ 22) showed an increased prevalence from 2017 to 2021: 15.2 % (95 %CI 14.2–16.3) to 17.0 % (95 %CI 15.8–18.1). Poor or very poor self-rated health decreased from 7.8 % (95 %CI 7.1–8.5) in 2017 to 5.1 % (95 %CI 4.5–5.7) in 2021, then increased to 8.8 % (95 %CI 8.1–9.5) in 2022. Supplementary analyses of these indicators according to sex and age group revealed some statistically significant differences for each year: higher prevalence of younger adults (16–24 years) with high or very high psychological distress compared to other age groups, and more adults aged ≥ 65 years rated their health as poor/very poor compared with the youngest group (Supplementary Fig. 2-3).

### 3.3 Trends before and during the COVID-19 pandemic

**Fig. 2:**
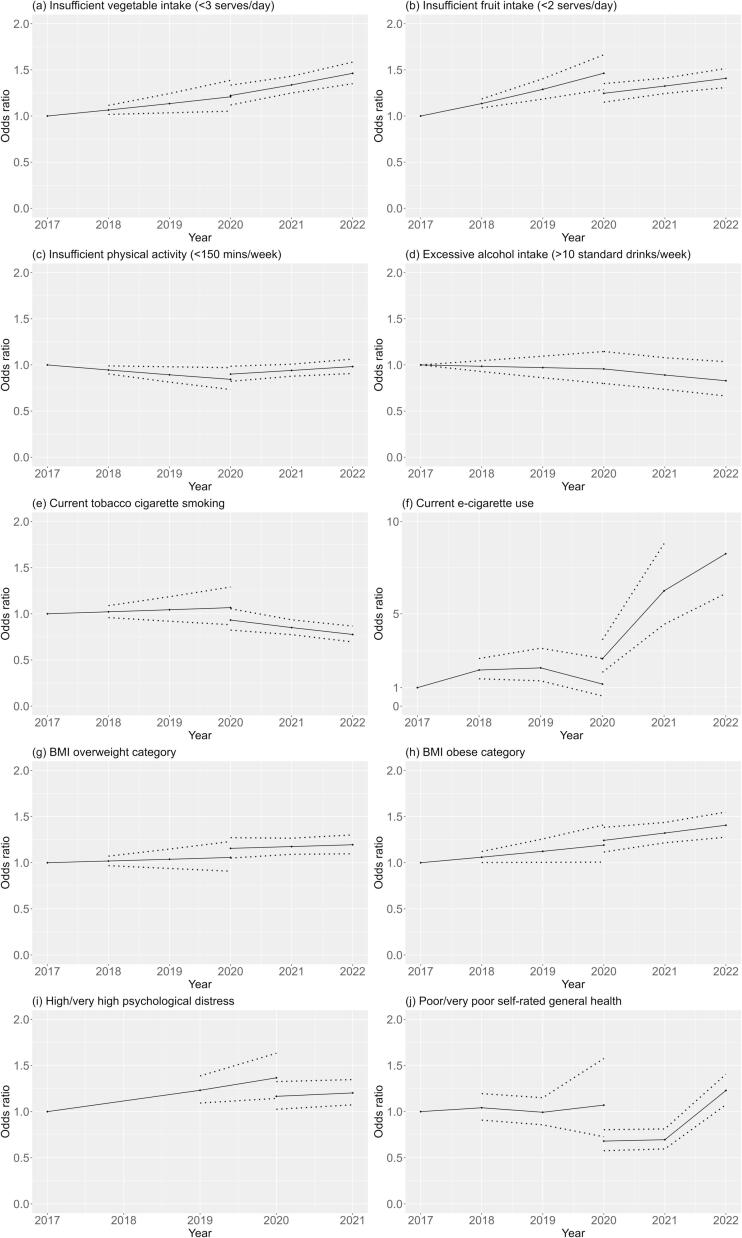
Interrupted time series models* for health behaviours, overweight, obesity, and wellbeing indicators among adults in New South Wales, Australia before (2017–2019) and during (2020–2022) the COVID-19 pandemic. (*Adjusted for age, sex, area-level socioeconomic status (SEIFA), remoteness (ARIA), education, language spoken, and country of birth based on Human Development Index.).

The interrupted time series models are illustrated in Fig. 2, with corresponding data in Supplementary Table 4.

Insufficient vegetable intake and insufficient vegetable intake both showed overall increasing trends, with statistically significant increases year-on-year compared to 2017. Insufficient fruit intake stabilised in 2020 (OR 1.25, 95 %CI 1.15–1.35) and 2021 (OR 1.32, 95 %CI 1.24–1.41), but in 2022 returned to pre-COVID levels (OR 1.41, 95 %CI 1.31–1.51). Tobacco smoking showed a stable trend during 2017–2020, with a significant decrease in 2021 (OR 0.85, 95 %CI 0.77–0.94) and 2022 (OR 0.78, 95 %CI 0.70–0.87). Conversely, e-cigarette use showed an overall increasing trend, with significant increases in 2021 (OR 6.25, 95 %CI 4.43–8.81) and 2022 (OR 8.25, 95 %CI 6.10–11.16) (i.e. the COVID years). Overall, during 2017–2022, BMI overweight and obese categories both showed steadily increasing trends, and insufficient physical activity and excessive alcohol intake were relatively stable.

For the wellbeing indicators investigated, changes were seen for self-rated health and psychological distress. High or very high psychological distress (K10 score ≥ 22) was relatively stable overall, though showed an increasing trend before COVID 2017–2020 and stabilising during COVID (2021 OR 1.20 95 %CI 1.07–1.35). Poor or very poor self-rated health showed a stable trend before COVID 2017–2020, but decreased in 2020 (OR 0.68, 95 %CI 0.58–0.80) and 2021 (OR 0.70, 95 %CI 0.60–0.81) (i.e., self-rated health *improved* during COVID years 2020–2021) then returned to pre-COVID levels in 2022 (OR 1.23, 95 %CI 1.07–1.41).

### 3.4 COVID-19-related trend changes by socioeconomic status

We investigated weighted prevalence trends (adjusted for covariates) according to SES indicators and education attainment (Supplementary Figures 4–6 and Supplementary Tables 5–7). Focusing on changes during the COVID-19 pandemic, there were few statistically significant changes in prevalence by SES indicators. Tobacco smoking prevalence showed a slight increase from 2020 to 2022 for the most disadvantaged group (SEIFA IRSD) (18.1 %, 95 %CI 14.4–21.8 to 19.8 % 95 %CI 16.8–22.8), compared to decreased trends among the least disadvantaged group (7.6 %, 95 %CI 5.8–9.3 to 6.2 %, 95 %CI 4.9–7.4). This was mirrored for tobacco smoking prevalence by education attainment categories. Tobacco smoking also decreased in major cities from 2020 to 2021 (13.1 %, 95 %CI 11.9–14.4 to 10.1 %, 95 %CI 10.1–11.8) compared to an increase in outer regional and remote areas (18.5 %, 95 %CI 12.7–24.3 to 20.2 %, 95 %CI 16.1–24.3). Insufficient physical activity prevalence increased from 2020 to 2021 for outer regional and remote areas (35.8 %, 95 %CI 29.6–42.0 to 36.4 %, 95 %CI 31.6–41.3) and for major cities and inner regional areas (31.2 %, 95 %CI 29.6–32.8 to 32.5 %, 95 %CI 31.2–33.8).

## 4 Discussion

This study is the first to present a trend analysis of health risk behaviours and wellbeing indicators using population-level data in Australia before and during the COVID-19 pandemic (2017–2022). The behavioural risk factor and overweight/obesity trends were mostly unchanged, as were wellbeing trends, with minor variations across age groups. Our findings are encouraging as the COVID-19 impact on chronic disease risk factors so far appears minimal at a population level in NSW, Australia. However, improved population rates of obesity and many health behaviours would support better overall health and wellbeing. Ongoing investment is warranted, particularly focused investment to improve equity.

While most risk factor and wellbeing indicators remained unchanged, we did see notable changes for e-cigarette use and self-rated health. Cigarette smoking showed some improvements in COVID years 2021–22, but for e-cigarette use there was a marked increase. The e-cigarette usage could be explained in part by an overall global trend toward increased e-cigarette usage in recent years (Freeman et al., 2022), however, when looking at population sub-groups in Australia, the increased usage during COVID is striking among young people (NSW Health, 2022, Watts et al., 2022 Dec). This rise in e-cigarette use is a threat to public health with increasing evidence of adverse health outcomes, including increased cigarette smoking among non-smokers, poisonings and exposure to harmful chemicals, and emerging evidence of longer-term chronic disease outcomes (Banks et al., 2023, Banks et al., 2023). Renewed tobacco control measures and investment in the regulation of e-cigarettes are essential as we redirect public health action beyond COVID years (Freeman, 2023). The Australian government is demonstrating such action through the recent endorsement of the National Tobacco Strategy 2023–2030 (Australian Government, 2023), and the introduction of new laws regulating e-cigarettes as of January 2024 (Australian Government, 2024).

Unexpectedly, we found that self-rated general health improved in 2020–21, yet in 2022 returned to pre-COVID levels. There are several possible explanations for this finding: social comparison (there were fewer cases in Australia compared to the world, so respondents were less likely to rate their health as poor), sample selection bias (those worried about being sick opted not to complete the surveys), and self-reporting bias (COVID-19 changed respondents internal frame for general health, and as a result, more reported feeling generally well). Perceptions of risk from COVID-19 are highly personal and influenced by contextual factors (e.g. media coverage, beliefs about personal health, previous experiences) (Lupton and Lewis, 2022), which could contribute to an individual’s improved sense of general health. Notably, we did identify age differences, with more young people rating health as poor/very poor or experiencing high/very high psychological distress, which is consistent with other research findings among young adults during the pandemic (Varma et al., 2021, Upton et al., 2021, Li et al., 2022, Zhao et al., 2022 Jun 15).

Our findings confirmed anticipated differences in health risk behaviours and wellbeing indicators by SES, but SES inequalities did not seem to be exacerbated during the COVID-19 pandemic (2020 to 2022). This finding was also somewhat surprising, given that people from major cities, lower socioeconomic groups and those born overseas are overrepresented in COVID-19 incidence and mortality rates in Australia (Australian Institute of Health and Welfare, 2022). Importantly, our population-level analysis is over a relatively short time period and does not investigate community sub-group or local-level differences, which could uncover differences in health behaviours and wellbeing not identified in this population-level analysis. For example, in 2021, community sub-group COVID-19 outbreaks were seen among culturally and linguistically diverse and lower-income communities living in localised areas; during this time, rapid, culturally appropriate responses were essential (Ioannides et al., 2022, Zachariah et al., 2022). Continued targeted support and sub-group monitoring of trends remain important for reducing health disparities and inequities.

### 4.1 Implications

These findings inform health policy and practices in Australia by providing data to understand the impact of the COVID-19 pandemic on population health behaviour and wellbeing. While our analysis suggests relatively minor population-level impacts of the COVID-19 pandemic on behaviours and wellbeing among NSW adults, continued surveillance of sub-group and individual-level differences is essential. This is especially relevant in Australia because 2022/2023 saw increased COVID-19 cases, reduced restrictions, increased vaccination rates, and changes to government economic support packages; as such, differences may yet emerge from regions that experienced greater lockdown measures or sub-groups that experienced greater impacts (e.g. those affected by periods of unemployment). Ongoing investment to improve population health and wellbeing is warranted, in addition to responsive, equitable targeting of health information, services, and programs.

### 4.2 Strengths and limitations

The analysis approach using ITS models is a strength and a unique analysis of health behaviours and wellbeing before and during the COVID-19 pandemic. There is limited sampling bias due to large sample sizes (>11,000 per survey), and limited effect of short-term fluctuations in COVID-19 cases and measures because surveys are generally conducted between February and December each year. This study uses self-reported measures of health behaviours and wellbeing indicators. While this is common in population health surveillance, it could introduce inaccurate or biased data. There were some notable differences in the 2021–2022 survey samples compared to 2017–2020, such as age, level of education, and some behavioural risk factors. These unweighted and unadjusted prevalence differences likely reflect the younger survey sample and the change to a mobile phone-based sampling frame in 2021. Using survey weights for population representativeness aims to overcome this in the analysis. We were unable to ascertain the month of data collection, which would have allowed for a more nuanced mapping against COVID-19 restrictions and cases in NSW. Some variables are not included in the NSW Adult Population Health Survey every year; so trend analyses for some variables of interest were not possible. Similarly, we were not able to use older data (before 2017) as there have been some changes to the survey items used prior to that time.

## 5 Conclusions

Before and during the COVID-19 pandemic (2017–2022), there was little change in behavioural risk factors (alcohol intake, smoking, physical activity, fruit, and vegetable intake), BMI category (overweight or obese) and wellbeing (psychological distress and self-rated general health) trends among adults in NSW, Australia. The trends showed limited variation by SES; however, established socioeconomic inequalities persisted, with the least advantaged generally with poorer risk factor levels and wellbeing. These findings highlight a limited population-level impact on health behaviours and wellbeing in NSW, Australia. Continued monitoring of risk factors during (and post) the COVID-19 pandemic is necessary, as is socioeconomic targeting of preventive health interventions.

## Funding

This work was supported by the Physical Activity Nutrition and Obesity Research Group and the Prevention Research Support Program, both funded by the New South Wales Ministry of Health. This study was completed while Christian Young was employed as a trainee on the NSW Biostatistics Training Program funded by the NSW Ministry of Health, and based at the Prevention Research Collaboration, University of Sydney.

## CRediT authorship contribution statement

**Sarah Marshall:** Writing – review & editing, Writing – original draft, Visualization, Conceptualization. **Bronwyn McGill:** Writing – review & editing, Writing – original draft, Visualization, Supervision, Project administration, Conceptualization. **Christian Young:** Writing – review & editing, Writing – original draft, Visualization, Methodology, Formal analysis. **Philip Clare:** Writing – review & editing, Writing – original draft, Visualization, Supervision, Methodology, Formal analysis, Conceptualization. **Sarah Neill:** Writing – review & editing, Writing – original draft, Visualization, Conceptualization. **Margaret Thomas:** Writing – review & editing, Conceptualization. **Adrian Bauman:** Writing – review & editing, Writing – original draft, Supervision, Methodology, Conceptualization.

## Declaration of competing interest

The authors declare that they have no known competing financial interests or personal relationships that could have appeared to influence the work reported in this paper.

## Data Availability

The authors do not have permission to share data.
