# Supplementary material for: Health behaviour and wellbeing trends among Australian adults before and during the COVID-19 pandemic (2017–2022): An interrupted time-series analysis
Source: Prev Med Rep. 2024 Aug 21;46:102861. doi: 10.1016/j.pmedr.2024.102861 (PMC11386313; doi:10.1016/j.pmedr.2024.102861)
Supplement: Supplementary Data 1 [file mmc1.pdf]

Supplementary Table 1

Table S1. NSW Population Health Survey variables (codes and labels) used in this study

Variable code	Variable Label
ALC1	ALC1 : How often have an alcoholic drink
ALC1a	ALC1a : How often in days per week have an alcoholic drink (NUM)
ALC2	ALC2 : Usual number of standard drinks per day
ALC2a	ALC2a : How many standard drinks per day (NUM)
ALC3	ALC3 : More than 4 male/2 female drinks in a day in past 4 weeks
ALC4	ALC4 : More than 11 male/7 female drinks in a day in past 4 weeks
ALC4a	ALC4a : More than 11 male/7 female drinks in a day in past 4 weeks number of tim
ALC5	ALC5 : 7-10 male/5-6 female drinks in a day in the past 4 weeks
ALC5a	ALC5a : 7-10 male/5-6 female drinks in a day in the past 4 weeks number of times
SMK1	SMK1 : Personal smoking status
SMKEL1	SMKEL1 : Current use of electronic cigarettes
HWT1	HWT1 : Height
HWT2	HWT2 : Weight
PAC1	PAC1 : Number of times walked in last week (NUM)
PAC2	PAC2 : Hours spent walking in last week (NUM)
PAC2b	PAC2b : Minutes spent walking in last week (NUM)
PAC7	PAC7 : Number of times exercised vigorously in last week (NUM)
PAC8a	PAC8a : Hours spent exercising vigorously in last week (NUM)
PAC8b	PAC8b : Minutes spent exercising vigorously in last week (NUM)
PAC9	PAC9 : Number of times spent exercising moderately in last week (NUM)
PAC10a	PAC10a : Hours spent exercising moderately in last week (NUM)
PAC10b	PAC10b : Minutes spent exercising moderately in last week (NUM)
NUT1	NUT1 : Serves of vegetables usually eaten per day
NUT1a	NUT1a : How many serves of vegetables usually eaten per day (NUM)
NUT1b	NUT1b : How many serves of vegetables usually eaten per week (NUM)
NUT2	NUT2 : Serves of fruit usually eaten per day
NUT2a	NUT2a : How many serves of fruit usually eaten per day (NUM)
NUT2b	NUT2b : How many serves of fruit usually eaten per week (NUM)
d_k10_score	K10 Score
HSD4	HSD4 : Self rated general health
disadvqt	SEIFA IRSD (2016)
ariaplusc	Remoteness quintile
QALLP	QALLP : Highest qualification completed
cob	Respondent's country of birth (Adult/Child)
LANPa	LANPa : Speak a language other than English at home
recnum	Unique ID
year	year
age	AGE :
sex	SEX : Respondent/Child sex
cob	Respondent's country of birth (Adult/Child)
wgt	WGT: Annual wgt

Supplementary Table 2

Table S2. Assessment of multicollinearity using variance inflation factors (VIF)

Covariate	VIF
Top SEIFA tertile	2.16
Middle SEIFA tertile	1.90
High school grade 12/TAFE/Diploma	1.95
University or greater	1.78
Language other than English spoken at home	1.51
Country of birth very high HDI	1.30
Country of birth high HDI	1.22
Outer regional/remote/very remote (ARIA+)	1.18
Age	1.18
Sex	1.01

Note: using a cut-point of 3

Supplementary Table 3

Table S3. Weighted prevalence of health risk behaviours, BMI category, and wellbeing indicators in NSW adults by year, 2017-2022

Outcome	Year	Prevalence	Standard Error	95% CI lower	95% CI upper
**Insufficient vegetable intake (<3 serves/day)**	2017	64.97%	0.67%	63.67%	66.28%
	2018	66.56%	0.67%	65.25%	67.88%
	2019	66.91%	0.75%	65.44%	68.38%
	2020	68.48%	0.81%	66.89%	70.07%
	2021	68.87%	0.66%	67.58%	70.15%
	2022	71.33%	0.62%	70.11%	72.55%
**Insufficient fruit intake (<2 serves/day)**	2017	53.65%	0.70%	52.28%	55.01%
	2018	59.05%	0.70%	57.68%	60.42%
	2019	59.48%	0.77%	57.96%	60.99%
	2020	59.70%	0.85%	58.04%	61.37%
	2021	59.36%	0.71%	57.97%	60.76%
	2022	62.35%	0.67%	61.04%	63.65%
**Insufficient physical activity (MVPA minutes/week)**					
<150 mins	2017	34.32%	0.67%	33.02%	35.63%
	2018	32.69%	0.68%	31.35%	34.02%
	2019	31.62%	0.75%	30.15%	33.09%
	2020	31.65%	0.82%	30.05%	33.25%
	2021	29.88%	0.68%	28.56%	31.21%
	2022	32.72%	0.65%	31.44%	34.01%
150 to 300 mins	2017	16.42%	0.53%	15.39%	17.46%
	2018	17.79%	0.57%	16.67%	18.91%
	2019	16.22%	0.60%	15.04%	17.39%
	2020	17.21%	0.68%	15.87%	18.55%
	2021	16.39%	0.54%	15.34%	17.45%
	2022	16.40%	0.52%	15.38%	17.42%
300+ mins	2017	49.26%	0.70%	47.88%	50.64%
	2018	49.53%	0.73%	48.09%	50.96%
	2019	52.16%	0.80%	50.59%	53.74%
	2020	51.14%	0.90%	49.37%	52.91%
	2021	53.72%	0.73%	52.30%	55.15%
	2022	50.88%	0.70%	49.50%	52.25%
**Excessive alcohol intake (>10 standard drinks/week)**	2017	13.84%	0.48%	12.91%	14.78%
	2018	14.25%	0.50%	13.27%	15.23%
	2019	13.78%	0.53%	12.74%	14.82%

	2020	15.00%	0.65%	13.74%	16.27%
	2021	14.69%	0.53%	13.65%	15.73%
	2022	13.67%	0.45%	12.78%	14.56%
**Current tobacco cigarette smoking**	2017	15.19%	0.53%	14.15%	16.24%
	2018	14.77%	0.53%	13.73%	15.81%
	2019	15.45%	0.60%	14.27%	16.63%
	2020	13.33%	0.62%	12.11%	14.55%
	2021	12.04%	0.44%	11.17%	12.91%
	2022	11.37%	0.42%	10.55%	12.20%
**Current e-cigarette use**	2017	1.02%	0.14%	0.74%	1.30%
	2018	1.49%	0.19%	1.12%	1.85%
	2019	1.93%	0.24%	1.46%	2.39%
	2020	2.06%	0.27%	1.54%	2.59%
	2021	5.46%	0.37%	4.73%	6.19%
	2022	6.37%	0.35%	5.69%	7.06%
**Body Mass Index (BMI) category**					
Underweight	2017	2.97%	0.24%	2.49%	3.45%
	2018	2.63%	0.23%	2.17%	3.09%
	2019	2.93%	0.28%	2.38%	3.48%
	2020	2.95%	0.33%	2.31%	3.59%
	2021	1.98%	0.23%	1.52%	2.44%
	2022	2.79%	0.24%	2.31%	3.26%
Healthy weight	2017	43.24%	0.71%	41.86%	44.62%
	2018	42.88%	0.72%	41.46%	44.29%
	2019	41.63%	0.80%	40.07%	43.19%
	2020	40.27%	0.88%	38.55%	41.99%
	2021	40.05%	0.72%	38.63%	41.46%
	2022	38.93%	0.68%	37.60%	40.26%
Overweight	2017	32.76%	0.66%	31.46%	34.05%
	2018	33.03%	0.68%	31.69%	34.36%
	2019	32.82%	0.75%	31.34%	34.30%
	2020	34.20%	0.83%	32.57%	35.83%
	2021	34.72%	0.67%	33.41%	36.03%
	2022	33.99%	0.65%	32.71%	35.27%
Obese	2017	21.03%	0.55%	19.95%	22.12%
	2018	21.46%	0.57%	20.35%	22.58%
	2019	22.62%	0.66%	21.33%	23.91%
	2020	22.58%	0.72%	21.16%	24.00%
	2021	23.25%	0.57%	22.13%	24.38%
	2022	24.29%	0.58%	23.15%	25.43%
**Poor or very poor self-rated general health**	2017	7.78%	0.35%	7.09%	8.47%
	2018	8.02%	0.36%	7.31%	8.73%

	2019	7.64%	0.40%	6.86%	8.41%
	2020	5.26%	0.34%	4.59%	5.93%
	2021	5.10%	0.30%	4.52%	5.69%
	2022	8.80%	0.38%	8.07%	9.54%
**High or very high psychological distress (K10 ≥22)**	2017	15.26%	0.53%	14.23%	16.29%
	2019	17.82%	0.64%	16.56%	19.08%
	2020	16.70%	0.69%	15.35%	18.06%
	2021	16.98%	0.59%	15.81%	18.14%

Supplementary Table 4

Table S4. Interrupted time series models for health behaviours, overweight, obesity, and wellbeing indicators in NSW adults before (2017-2019) and during (2020-2022) the COVID-19 pandemic

Outcome		Odds ratio	95% CI (lower)	95%CI (upper)	*p-value*
**Insufficient vegetable intake (<3 serves/day)**					
Pre COVID trend					
	2017	*ref*			
	2018	1.07	1.02	1.12	0.007
	2019	1.13	1.03	1.24	0.007
	2020	1.21	1.05	1.39	0.007
During COVID trend					
	2020	1.22	1.12	1.33	<0.001
	2021	1.34	1.25	1.43	<0.001
	2022	1.46	1.35	1.58	<0.001
**Insufficient fruit intake (<2 serves/day)**					
Pre COVID trend					
	2017	*ref*			
	2018	1.14	1.09	1.18	<0.001
	2019	1.29	1.18	1.40	<0.001
	2020	1.46	1.29	1.66	<0.001
During COVID trend					
	2020	1.25	1.15	1.35	<0.001
	2021	1.32	1.24	1.41	<0.001
	2022	1.41	1.31	1.51	<0.001
**Insufficient physical activity (<150 MVPA minutes/week)**					
Pre COVID trend					
	2017	*ref*			
	2018	0.94	0.90	0.99	0.017
	2019	0.89	0.81	0.98	0.017
	2020	0.84	0.73	0.97	0.017
During COVID trend					
	2020	0.90	0.82	0.99	0.023
	2021	0.94	0.88	1.01	0.080
	2022	0.98	0.91	1.06	0.653
**Excessive alcohol intake (>10 standard drinks/week)**					
Pre COVID trend					
	2017	*ref*			
	2018	0.99	0.93	1.05	0.628

	2019	0.97	0.86	1.09	0.628
	2020	0.96	0.80	1.15	0.628
During COVID trend					
	2020	0.96	0.80	1.15	0.628
	2021	0.89	0.74	1.08	0.234
	2022	0.83	0.66	1.04	0.098
**Current tobacco cigarette smoking**					
Pre COVID trend					
	2017	*ref*			
	2018	1.02	0.96	1.09	0.504
	2019	1.04	0.92	1.19	0.504
	2020	1.07	0.88	1.29	0.504
During COVID trend					
	2020	0.93	0.82	1.06	0.268
	2021	0.85	0.77	0.94	0.001
	2022	0.78	0.70	0.87	<0.001
**Current e-cigarette use**					
Pre COVID trend					
	2017				
	2018	1.95	1.48	2.58	<0.001
	2019	2.07	1.37	3.14	0.001
	2020	1.19	0.55	2.58	0.654
During COVID trend					
	2020	2.57	1.83	3.59	<0.001
	2021	6.25	4.43	8.81	<0.001
	2022	8.25	6.10	11.16	<0.001
**Body Mass Index (BMI) category**					
**Underweight**					
Pre COVID trend					
	2017				
	2018	1.02	0.89	1.17	0.760
	2019	1.04	0.80	1.37	0.760
	2020	1.07	0.71	1.60	0.760
During COVID trend					
	2020	1.00	0.76	1.32	0.982
	2021	1.00	0.82	1.23	0.967
	2022	1.01	0.79	1.29	0.966
**Overweight**					
Pre COVID trend					
	2017				
	2018	1.02	0.97	1.07	0.474
	2019	1.04	0.94	1.15	0.474
	2020	1.06	0.91	1.23	0.474

During COVID trend		1.00	1.00	1.00	
	2020	1.16	1.05	1.27	0.003
	2021	1.18	1.09	1.27	<0.001
	2022	1.19	1.10	1.30	<0.001
**Obese**					
Pre COVID trend					
	2017				
	2018	1.06	1.00	1.12	0.042
	2019	1.12	1.00	1.26	0.042
	2020	1.19	1.01	1.41	0.042
During COVID trend		1.00	1.00	1.00	
	2020	1.24	1.12	1.38	<0.001
	2021	1.32	1.22	1.44	<0.001
	2022	1.41	1.28	1.55	<0.001
**Poor or very poor self-rated general health**					
Pre COVID trend					
	2017				
	2018	1.04	0.91	1.20	0.559
	2019	0.99	0.86	1.15	0.925
	2020	1.07	0.73	1.58	0.730
During COVID trend					
	2020	0.68	0.58	0.80	<0.001
	2021	0.70	0.60	0.81	<0.001
	2022	1.23	1.07	1.41	0.003
**High or very high psychological distress (K10 ≥22)**					
Pre COVID trend					
	2017				
	2019	1.23	1.09	1.39	0.001
	2020	1.37	1.14	1.63	0.001
During COVID trend					
	2020	1.17	1.03	1.33	0.019
	2021	1.20	1.07	1.35	0.001

Supplementary Table 5

Table S5. Weighted prevalence of health risk behaviours, BMI category, and wellbeing indicators in NSW adults by SEIFA, by year, 2017-2022

Outcome	Year	SEIFA category	Prevalence (%)	Standard Error	95% CI (lower)	95%CI (upper)
Alcohol (11+ serves of alcohol p/w)	2017	1 (More advantaged)	16.07%	1.10%	13.92%	18.22%
	2017	2-4	13.57%	0.57%	12.46%	14.68%
	2017	5 (More disadvantaged)	12.76%	1.08%	10.65%	14.87%
	2018	1 (More advantaged)	16.94%	1.10%	14.79%	19.10%
	2018	2-4	14.03%	0.61%	12.83%	15.22%
	2018	5 (More disadvantaged)	12.86%	1.12%	10.67%	15.06%
	2019	1 (More advantaged)	16.79%	1.25%	14.35%	19.23%
	2019	2-4	12.84%	0.60%	11.67%	14.01%
	2019	5 (More disadvantaged)	12.91%	1.21%	10.54%	15.29%
	2020	1 (More advantaged)	15.46%	1.16%	13.19%	17.74%
	2020	2-4	15.40%	0.83%	13.77%	17.02%
	2020	5 (More disadvantaged)	12.46%	1.53%	9.46%	15.45%
	2021	1 (More advantaged)	17.80%	1.43%	15.00%	20.60%
	2021	2-4	13.46%	0.52%	12.43%	14.48%
	2021	5 (More disadvantaged)	13.33%	1.15%	11.07%	15.59%
	2022	1 (More advantaged)	13.60%	0.92%	11.79%	15.40%
	2022	2-4	13.55%	0.54%	12.49%	14.61%
	2022	5 (More disadvantaged)	12.91%	1.18%	10.60%	15.22%
Current smoker	2017	1 (More advantaged)	10.67%	1.02%	8.67%	12.68%
	2017	2-4	15.34%	0.67%	14.02%	16.65%
	2017	5 (More disadvantaged)	20.06%	1.39%	17.33%	22.79%
	2018	1 (More advantaged)	9.67%	0.92%	7.87%	11.47%
	2018	2-4	15.24%	0.68%	13.90%	16.58%
	2018	5 (More disadvantaged)	19.63%	1.41%	16.86%	22.39%
	2019	1 (More advantaged)	7.87%	0.91%	6.09%	9.66%
	2019	2-4	15.66%	0.76%	14.16%	17.16%
	2019	5 (More disadvantaged)	24.17%	1.76%	20.73%	27.62%
	2020	1 (More advantaged)	7.55%	0.89%	5.80%	9.31%
	2020	2-4	14.62%	0.83%	12.99%	16.25%
	2020	5 (More disadvantaged)	18.02%	1.87%	14.36%	21.68%
	2021	1 (More advantaged)	7.91%	0.80%	6.34%	9.47%
	2021	2-4	11.96%	0.54%	10.90%	13.02%
	2021	5 (More disadvantaged)	19.46%	1.43%	16.65%	22.27%
	2022	1 (More advantaged)	6.16%	0.65%	4.89%	7.43%
	2022	2-4	11.55%	0.52%	10.53%	12.56%
	2022	5 (More disadvantaged)	19.83%	1.54%	16.82%	22.84%
Current eCigarette user	2017	1 (More advantaged)	0.87%	0.30%	0.28%	1.46%
	2017	2-4	1.09%	0.18%	0.73%	1.45%

	2017	5 (More disadvantaged)	0.95%	0.36%	0.24%	1.66%
	2018	1 (More advantaged)	1.30%	0.42%	0.48%	2.12%
	2018	2-4	1.41%	0.22%	0.97%	1.84%
	2018	5 (More disadvantaged)	2.05%	0.56%	0.95%	3.15%
	2019	1 (More advantaged)	2.42%	0.59%	1.27%	3.57%
	2019	2-4	1.79%	0.28%	1.24%	2.34%
	2019	5 (More disadvantaged)	1.95%	0.63%	0.71%	3.18%
	2020	1 (More advantaged)	2.56%	0.68%	1.23%	3.89%
	2020	2-4	2.02%	0.32%	1.39%	2.64%
	2020	5 (More disadvantaged)	1.59%	0.61%	0.40%	2.79%
	2021	1 (More advantaged)	4.52%	0.66%	3.22%	5.83%
	2021	2-4	5.32%	0.45%	4.45%	6.19%
	2021	5 (More disadvantaged)	6.65%	0.99%	4.70%	8.60%
	2022	1 (More advantaged)	5.97%	0.72%	4.56%	7.38%
	2022	2-4	6.74%	0.44%	5.88%	7.60%
	2022	5 (More disadvantaged)	5.63%	0.85%	3.96%	7.29%
BMI (healthy weight)	2017	1 (More advantaged)	51.84%	1.54%	48.83%	54.85%
	2017	2-4	41.15%	0.87%	39.44%	42.85%
	2017	5 (More disadvantaged)	39.38%	1.61%	36.23%	42.54%
	2018	1 (More advantaged)	51.13%	1.51%	48.17%	54.09%
	2018	2-4	42.03%	0.89%	40.28%	43.78%
	2018	5 (More disadvantaged)	34.69%	1.65%	31.46%	37.92%
	2019	1 (More advantaged)	48.18%	1.71%	44.83%	51.53%
	2019	2-4	41.70%	0.98%	39.77%	43.62%
	2019	5 (More disadvantaged)	34.19%	1.80%	30.67%	37.72%
	2020	1 (More advantaged)	48.90%	1.68%	45.61%	52.20%
	2020	2-4	38.94%	1.09%	36.81%	41.07%
	2020	5 (More disadvantaged)	31.75%	2.17%	27.49%	36.00%
	2021	1 (More advantaged)	47.91%	1.52%	44.93%	50.90%
	2021	2-4	38.10%	0.85%	36.44%	39.76%
	2021	5 (More disadvantaged)	33.37%	1.71%	30.03%	36.72%
	2022	1 (More advantaged)	47.45%	1.43%	44.65%	50.24%
	2022	2-4	37.58%	0.81%	35.99%	39.18%
	2022	5 (More disadvantaged)	31.30%	1.72%	27.92%	34.67%
BMI (underweight)	2017	1 (More advantaged)	3.23%	0.59%	2.07%	4.38%
	2017	2-4	3.34%	0.32%	2.71%	3.97%
	2017	5 (More disadvantaged)	1.40%	0.39%	0.63%	2.17%
	2018	1 (More advantaged)	3.02%	0.51%	2.03%	4.01%
	2018	2-4	2.49%	0.29%	1.92%	3.06%
	2018	5 (More disadvantaged)	2.44%	0.55%	1.36%	3.52%
	2019	1 (More advantaged)	3.05%	0.59%	1.89%	4.20%
	2019	2-4	2.78%	0.33%	2.13%	3.43%
	2019	5 (More disadvantaged)	3.29%	0.75%	1.82%	4.77%
	2020	1 (More advantaged)	3.09%	0.59%	1.93%	4.25%
	2020	2-4	3.11%	0.43%	2.27%	3.95%
	2020	5 (More disadvantaged)	2.20%	0.82%	0.60%	3.80%

	2021	1 (More advantaged)	1.56%	0.55%	0.49%	2.63%
	2021	2-4	2.03%	0.27%	1.49%	2.57%
	2021	5 (More disadvantaged)	2.55%	0.55%	1.47%	3.62%
	2022	1 (More advantaged)	3.79%	0.57%	2.67%	4.92%
	2022	2-4	2.09%	0.25%	1.61%	2.57%
	2022	5 (More disadvantaged)	4.01%	0.81%	2.42%	5.61%
BMI (overweight)	2017	1 (More advantaged)	31.94%	1.44%	29.13%	34.76%
	2017	2-4	33.08%	0.84%	31.44%	34.72%
	2017	5 (More disadvantaged)	32.86%	1.54%	29.84%	35.87%
	2018	1 (More advantaged)	32.52%	1.42%	29.74%	35.31%
	2018	2-4	33.37%	0.85%	31.70%	35.04%
	2018	5 (More disadvantaged)	32.67%	1.64%	29.45%	35.89%
	2019	1 (More advantaged)	33.84%	1.61%	30.68%	36.99%
	2019	2-4	32.81%	0.93%	30.99%	34.63%
	2019	5 (More disadvantaged)	31.09%	1.82%	27.52%	34.65%
	2020	1 (More advantaged)	34.54%	1.57%	31.46%	37.62%
	2020	2-4	34.64%	1.05%	32.57%	36.71%
	2020	5 (More disadvantaged)	31.03%	2.08%	26.95%	35.12%
	2021	1 (More advantaged)	34.86%	1.41%	32.09%	37.63%
	2021	2-4	34.66%	0.81%	33.08%	36.24%
	2021	5 (More disadvantaged)	34.92%	1.74%	31.51%	38.34%
	2022	1 (More advantaged)	33.77%	1.30%	31.22%	36.31%
	2022	2-4	34.24%	0.81%	32.66%	35.82%
	2022	5 (More disadvantaged)	32.32%	1.77%	28.85%	35.79%
BMI (Obese)	2017	1 (More advantaged)	12.99%	1.00%	11.03%	14.95%
	2017	2-4	22.43%	0.71%	21.03%	23.83%
	2017	5 (More disadvantaged)	26.35%	1.41%	23.60%	29.11%
	2018	1 (More advantaged)	13.32%	1.01%	11.35%	15.30%
	2018	2-4	22.11%	0.72%	20.69%	23.53%
	2018	5 (More disadvantaged)	30.20%	1.52%	27.22%	33.19%
	2019	1 (More advantaged)	14.94%	1.23%	12.54%	17.34%
	2019	2-4	22.71%	0.81%	21.12%	24.31%
	2019	5 (More disadvantaged)	31.43%	1.77%	27.96%	34.89%
	2020	1 (More advantaged)	13.47%	1.07%	11.38%	15.56%
	2020	2-4	23.31%	0.91%	21.54%	25.09%
	2020	5 (More disadvantaged)	35.02%	2.28%	30.55%	39.49%
	2021	1 (More advantaged)	15.67%	1.00%	13.71%	17.63%
	2021	2-4	25.21%	0.72%	23.80%	26.62%
	2021	5 (More disadvantaged)	29.16%	1.67%	25.89%	32.42%
	2022	1 (More advantaged)	14.99%	1.01%	13.02%	16.97%
	2022	2-4	26.09%	0.74%	24.63%	27.54%
	2022	5 (More disadvantaged)	32.37%	1.75%	28.94%	35.79%
<3 serves of vegetables p/d	2017	1 (More advantaged)	58.15%	1.48%	55.25%	61.05%
	2017	2-4	65.62%	0.81%	64.03%	67.22%
	2017	5 (More disadvantaged)	69.91%	1.49%	67.00%	72.82%

	2018	1 (More advantaged)	60.84%	1.47%	57.97%	63.72%
	2018	2-4	66.73%	0.83%	65.10%	68.37%
	2018	5 (More disadvantaged)	71.68%	1.54%	68.66%	74.70%
	2019	1 (More advantaged)	60.79%	1.69%	57.49%	64.10%
	2019	2-4	67.95%	0.89%	66.19%	69.70%
	2019	5 (More disadvantaged)	71.65%	1.77%	68.19%	75.12%
	2020	1 (More advantaged)	63.42%	1.55%	60.38%	66.47%
	2020	2-4	68.98%	1.01%	67.00%	70.95%
	2020	5 (More disadvantaged)	74.71%	2.16%	70.48%	78.94%
	2021	1 (More advantaged)	63.68%	1.47%	60.79%	66.57%
	2021	2-4	70.14%	0.77%	68.64%	71.65%
	2021	5 (More disadvantaged)	75.29%	1.58%	72.20%	78.38%
	2022	1 (More advantaged)	64.34%	1.36%	61.67%	67.01%
	2022	2-4	72.91%	0.74%	71.47%	74.36%
	2022	5 (More disadvantaged)	77.93%	1.56%	74.87%	80.99%
<2 serves of fruit p/d	2017	1 (More advantaged)	48.09%	1.55%	45.06%	51.12%
	2017	2-4	55.39%	0.88%	53.67%	57.10%
	2017	5 (More disadvantaged)	54.13%	1.67%	50.86%	57.41%
	2018	1 (More advantaged)	54.99%	1.55%	51.96%	58.02%
	2018	2-4	59.77%	0.89%	58.03%	61.50%
	2018	5 (More disadvantaged)	61.73%	1.64%	58.51%	64.95%
	2019	1 (More advantaged)	54.96%	1.69%	51.64%	58.28%
	2019	2-4	60.02%	0.97%	58.11%	61.93%
	2019	5 (More disadvantaged)	63.40%	1.89%	59.70%	67.09%
	2020	1 (More advantaged)	58.33%	1.65%	55.09%	61.57%
	2020	2-4	59.92%	1.10%	57.77%	62.08%
	2020	5 (More disadvantaged)	61.30%	2.33%	56.73%	65.86%
	2021	1 (More advantaged)	55.48%	1.63%	52.29%	58.67%
	2021	2-4	60.13%	0.85%	58.47%	61.78%
	2021	5 (More disadvantaged)	62.15%	1.77%	58.68%	65.62%
	2022	1 (More advantaged)	57.65%	1.44%	54.83%	60.47%
	2022	2-4	63.11%	0.82%	61.51%	64.71%
	2022	5 (More disadvantaged)	67.48%	1.76%	64.04%	70.93%
<150 mins of moderate/vigorous activity p/w	2017	1 (More advantaged)	24.43%	1.25%	21.98%	26.89%
	2017	2-4	35.99%	0.84%	34.35%	37.64%
	2017	5 (More disadvantaged)	39.47%	1.53%	36.47%	42.48%
	2018	1 (More advantaged)	21.37%	1.15%	19.11%	23.63%
	2018	2-4	34.15%	0.87%	32.44%	35.87%
	2018	5 (More disadvantaged)	39.93%	1.65%	36.69%	43.17%
	2019	1 (More advantaged)	24.90%	1.43%	22.09%	27.71%
	2019	2-4	31.94%	0.92%	30.14%	33.73%
	2019	5 (More disadvantaged)	38.38%	1.83%	34.79%	41.96%
	2020	1 (More advantaged)	25.00%	1.49%	22.08%	27.93%
	2020	2-4	31.84%	1.00%	29.88%	33.80%

	2020	5 (More disadvantaged)	40.64%	2.37%	36.00%	45.28%
	2021	1 (More advantaged)	23.55%	1.64%	20.34%	26.76%
	2021	2-4	31.16%	0.80%	29.60%	32.72%
	2021	5 (More disadvantaged)	39.49%	1.79%	35.97%	43.00%
	2022	1 (More advantaged)	24.64%	1.18%	22.33%	26.95%
	2022	2-4	33.92%	0.82%	32.32%	35.53%
	2022	5 (More disadvantaged)	41.42%	1.81%	37.87%	44.97%
150 -300 mins of moderate/vigorous activity p/w	2017	1 (More advantaged)	16.02%	1.12%	13.82%	18.22%
	2017	2-4	17.26%	0.69%	15.91%	18.62%
	2017	5 (More disadvantaged)	14.04%	1.14%	11.82%	16.27%
	2018	1 (More advantaged)	16.94%	1.14%	14.71%	19.18%
	2018	2-4	17.78%	0.74%	16.33%	19.24%
	2018	5 (More disadvantaged)	18.79%	1.36%	16.12%	21.46%
	2019	1 (More advantaged)	15.06%	1.21%	12.68%	17.44%
	2019	2-4	16.01%	0.74%	14.57%	17.45%
	2019	5 (More disadvantaged)	18.38%	1.59%	15.26%	21.50%
	2020	1 (More advantaged)	16.25%	1.23%	13.83%	18.67%
	2020	2-4	18.19%	0.93%	16.37%	20.01%
	2020	5 (More disadvantaged)	14.63%	1.65%	11.39%	17.87%
	2021	1 (More advantaged)	17.03%	1.22%	14.63%	19.43%
	2021	2-4	16.34%	0.65%	15.07%	17.61%
	2021	5 (More disadvantaged)	16.01%	1.35%	13.36%	18.66%
	2022	1 (More advantaged)	16.51%	1.09%	14.37%	18.66%
	2022	2-4	16.65%	0.65%	15.38%	17.92%
	2022	5 (More disadvantaged)	15.38%	1.36%	12.71%	18.05%
>300 mins of moderate/vigorous activity p/w	2017	1 (More advantaged)	59.54%	1.47%	56.65%	62.43%
	2017	2-4	46.74%	0.88%	45.03%	48.46%
	2017	5 (More disadvantaged)	46.48%	1.63%	43.30%	49.67%
	2018	1 (More advantaged)	61.68%	1.41%	58.91%	64.46%
	2018	2-4	48.06%	0.92%	46.26%	49.87%
	2018	5 (More disadvantaged)	41.28%	1.70%	37.96%	44.60%
	2019	1 (More advantaged)	60.04%	1.62%	56.86%	63.22%
	2019	2-4	52.05%	0.98%	50.13%	53.97%
	2019	5 (More disadvantaged)	43.24%	1.94%	39.44%	47.05%
	2020	1 (More advantaged)	58.74%	1.66%	55.48%	62.00%
	2020	2-4	49.97%	1.13%	47.76%	52.19%
	2020	5 (More disadvantaged)	44.72%	2.33%	40.15%	49.30%
	2021	1 (More advantaged)	59.42%	1.72%	56.06%	62.79%
	2021	2-4	52.50%	0.86%	50.82%	54.19%
	2021	5 (More disadvantaged)	44.50%	1.79%	40.99%	48.01%
	2022	1 (More advantaged)	58.85%	1.37%	56.16%	61.54%
	2022	2-4	49.43%	0.85%	47.76%	51.09%
	2022	5 (More disadvantaged)	43.20%	1.86%	39.56%	46.84%

K10 scores 22+	2017	1 (More advantaged)	10.28%	0.94%	8.44%	12.12%
	2017	2-4	15.83%	0.68%	14.49%	17.16%
	2017	5 (More disadvantaged)	19.18%	1.35%	16.53%	21.82%
	2018	1 (More advantaged)	13.98%	1.27%	11.48%	16.47%
	2018	2-4	17.84%	0.80%	16.28%	19.40%
	2018	5 (More disadvantaged)	22.66%	1.72%	19.29%	26.03%
	2019	1 (More advantaged)	14.47%	1.35%	11.83%	17.11%
	2019	2-4	17.11%	0.87%	15.42%	18.81%
	2019	5 (More disadvantaged)	19.47%	1.94%	15.66%	23.28%
	2020	1 (More advantaged)	14.87%	1.42%	12.09%	17.65%
	2020	2-4	16.83%	0.64%	15.58%	18.08%
	2020	5 (More disadvantaged)	20.61%	1.48%	17.70%	23.52%
Poor/very poor health	2017	1 (More advantaged)	5.15%	0.63%	3.92%	6.38%
	2017	2-4	8.05%	0.46%	7.15%	8.95%
	2017	5 (More disadvantaged)	10.00%	0.88%	8.27%	11.72%
	2018	1 (More advantaged)	5.02%	0.65%	3.75%	6.30%
	2018	2-4	8.23%	0.47%	7.31%	9.16%
	2018	5 (More disadvantaged)	10.77%	0.95%	8.91%	12.63%
	2019	1 (More advantaged)	5.42%	0.72%	4.00%	6.83%
	2019	2-4	7.73%	0.50%	6.74%	8.72%
	2019	5 (More disadvantaged)	9.85%	1.07%	7.76%	11.94%
	2020	1 (More advantaged)	3.41%	0.64%	2.15%	4.67%
	2020	2-4	5.62%	0.44%	4.76%	6.48%
	2020	5 (More disadvantaged)	6.66%	0.93%	4.84%	8.48%
	2021	1 (More advantaged)	3.08%	0.53%	2.05%	4.11%
	2021	2-4	5.72%	0.39%	4.96%	6.47%
	2021	5 (More disadvantaged)	6.79%	0.95%	4.93%	8.64%
	2022	1 (More advantaged)	6.64%	0.66%	5.34%	7.94%
	2022	2-4	9.09%	0.47%	8.16%	10.01%
	2022	5 (More disadvantaged)	11.24%	1.16%	8.96%	13.52%

Supplementary Table 6

Table S6. Weighted prevalence of health risk behaviours, BMI category, and wellbeing indicators in NSW adults by ARIA, by year, 2017-2022

Outcome	Year	ARIA category	Prevalence (%)	Standard Error	95% CI (lower)	95%CI (upper)
Alcohol (11+ serves of alcohol p/w)	2017	Metro & inner regional	13.88%	0.47%	12.95%	14.81%
	2017	Outer regional & remote	15.34%	1.88%	11.65%	19.02%
	2018	Metro & inner regional	14.51%	0.50%	13.53%	15.50%
	2018	Outer regional & remote	13.92%	1.66%	10.66%	17.18%
	2019	Metro & inner regional	13.49%	0.51%	12.48%	14.50%
	2019	Outer regional & remote	17.06%	2.19%	12.76%	21.35%
	2020	Metro & inner regional	14.96%	0.65%	13.69%	16.23%
	2020	Outer regional & remote	16.13%	2.19%	11.83%	20.43%
	2021	Metro & inner regional	14.40%	0.54%	13.34%	15.46%
	2021	Outer regional & remote	17.51%	1.72%	14.14%	20.87%
	2022	Metro & inner regional	13.21%	0.45%	12.33%	14.08%
	2022	Outer regional & remote	17.66%	1.80%	14.13%	21.19%
Current smoker	2017	Metro & inner regional	14.80%	0.53%	13.76%	15.85%
	2017	Outer regional & remote	22.69%	2.84%	17.14%	28.25%
	2018	Metro & inner regional	14.65%	0.54%	13.59%	15.71%
	2018	Outer regional & remote	17.53%	2.28%	13.06%	21.99%
	2019	Metro & inner regional	15.43%	0.62%	14.21%	16.65%
	2019	Outer regional & remote	16.51%	2.21%	12.18%	20.84%
	2020	Metro & inner regional	13.12%	0.63%	11.87%	14.36%
	2020	Outer regional & remote	18.41%	2.93%	12.66%	24.16%
	2021	Metro & inner regional	11.45%	0.44%	10.59%	12.32%
	2021	Outer regional & remote	21.14%	2.34%	16.57%	25.72%
	2022	Metro & inner regional	10.93%	0.43%	10.09%	11.77%
	2022	Outer regional & remote	20.19%	2.08%	16.11%	24.28%
Current eCigarette user	2017	Metro & inner regional	1.05%	0.15%	0.75%	1.34%
	2017	Outer regional & remote	0.47%	0.19%	0.09%	0.85%
	2018	Metro & inner regional	1.52%	0.20%	1.13%	1.90%
	2018	Outer regional & remote	1.00%	0.49%	0.04%	1.96%
	2019	Metro & inner regional	1.98%	0.25%	1.49%	2.47%
	2019	Outer regional & remote	1.35%	0.59%	0.19%	2.50%
	2020	Metro & inner regional	2.15%	0.28%	1.59%	2.70%
	2020	Outer regional & remote	0.92%	0.55%	-0.15%	2.00%
	2021	Metro & inner regional	5.38%	0.36%	4.68%	6.09%
	2021	Outer regional & remote	3.22%	1.00%	1.26%	5.18%
	2022	Metro & inner regional	6.51%	0.36%	5.81%	7.20%
	2022	Outer regional & remote	4.15%	1.17%	1.86%	6.44%

BMI (healthy weight)	2017	Metro & inner regional	43.38%	0.71%	41.99%	44.76%
	2017	Outer regional & remote	38.36%	2.97%	32.55%	44.18%
	2018	Metro & inner regional	43.19%	0.72%	41.78%	44.61%
	2018	Outer regional & remote	32.95%	2.56%	27.93%	37.96%
	2019	Metro & inner regional	42.28%	0.81%	40.69%	43.86%
	2019	Outer regional & remote	32.52%	2.81%	27.02%	38.03%
	2020	Metro & inner regional	40.81%	0.88%	39.08%	42.53%
	2020	Outer regional & remote	32.07%	3.30%	25.59%	38.55%
	2021	Metro & inner regional	40.33%	0.72%	38.92%	41.74%
	2021	Outer regional & remote	33.12%	2.64%	27.94%	38.30%
	2022	Metro & inner regional	39.28%	0.69%	37.94%	40.63%
	2022	Outer regional & remote	35.34%	2.71%	30.03%	40.66%
BMI (underweight)	2017	Metro & inner regional	3.01%	0.25%	2.52%	3.50%
	2017	Outer regional & remote	1.40%	0.52%	0.38%	2.42%
	2018	Metro & inner regional	2.60%	0.24%	2.13%	3.07%
	2018	Outer regional & remote	2.72%	0.87%	1.02%	4.42%
	2019	Metro & inner regional	2.96%	0.28%	2.40%	3.51%
	2019	Outer regional & remote	2.57%	1.01%	0.59%	4.55%
	2020	Metro & inner regional	3.00%	0.33%	2.35%	3.66%
	2020	Outer regional & remote	2.42%	0.94%	0.58%	4.25%
	2021	Metro & inner regional	1.98%	0.24%	1.52%	2.44%
	2021	Outer regional & remote	2.09%	0.99%	0.16%	4.03%
	2022	Metro & inner regional	2.76%	0.25%	2.28%	3.25%
	2022	Outer regional & remote	3.89%	1.23%	1.49%	6.29%
BMI (overweight)	2017	Metro & inner regional	32.85%	0.67%	31.53%	34.17%
	2017	Outer regional & remote	32.68%	2.55%	27.68%	37.68%
	2018	Metro & inner regional	33.30%	0.69%	31.94%	34.65%
	2018	Outer regional & remote	30.40%	2.45%	25.60%	35.21%
	2019	Metro & inner regional	32.71%	0.76%	31.21%	34.21%
	2019	Outer regional & remote	34.39%	3.00%	28.50%	40.27%
	2020	Metro & inner regional	34.41%	0.84%	32.76%	36.06%
	2020	Outer regional & remote	29.83%	3.06%	23.83%	35.82%
	2021	Metro & inner regional	34.84%	0.68%	33.51%	36.16%
	2021	Outer regional & remote	34.09%	2.44%	29.32%	38.87%
	2022	Metro & inner regional	33.92%	0.66%	32.62%	35.22%
	2022	Outer regional & remote	33.83%	2.49%	28.95%	38.70%
BMI (Obese)	2017	Metro & inner regional	20.76%	0.56%	19.66%	21.87%
	2017	Outer regional & remote	27.56%	2.54%	22.58%	32.54%
	2018	Metro & inner regional	20.91%	0.58%	19.78%	22.04%
	2018	Outer regional & remote	33.93%	2.57%	28.89%	38.97%
	2019	Metro & inner regional	22.06%	0.66%	20.75%	23.36%
	2019	Outer regional & remote	30.52%	2.86%	24.91%	36.12%
	2020	Metro & inner regional	21.78%	0.73%	20.36%	23.21%
	2020	Outer regional & remote	35.68%	3.36%	29.09%	42.28%
	2021	Metro & inner regional	22.85%	0.58%	21.72%	23.99%

	2021	Outer regional & remote	30.69%	2.46%	25.88%	35.50%
	2022	Metro & inner regional	24.03%	0.60%	22.85%	25.21%
	2022	Outer regional & remote	26.94%	2.12%	22.78%	31.10%
<3 serves of vegetables p/d	2017	Metro & inner regional	64.56%	0.67%	63.25%	65.87%
	2017	Outer regional & remote	68.66%	2.34%	64.07%	73.25%
	2018	Metro & inner regional	66.17%	0.68%	64.84%	67.50%
	2018	Outer regional & remote	68.36%	2.36%	63.73%	72.99%
	2019	Metro & inner regional	66.83%	0.76%	65.34%	68.31%
	2019	Outer regional & remote	70.35%	2.46%	65.53%	75.17%
	2020	Metro & inner regional	68.43%	0.82%	66.82%	70.03%
	2020	Outer regional & remote	68.11%	3.00%	62.23%	73.99%
	2021	Metro & inner regional	68.95%	0.66%	67.65%	70.25%
	2021	Outer regional & remote	72.10%	2.14%	67.90%	76.30%
	2022	Metro & inner regional	71.25%	0.63%	70.01%	72.49%
	2022	Outer regional & remote	74.80%	2.00%	70.88%	78.71%
<2 serves of fruit p/d	2017	Metro & inner regional	53.56%	0.72%	52.15%	54.96%
	2017	Outer regional & remote	54.94%	2.85%	49.35%	60.53%
	2018	Metro & inner regional	59.10%	0.72%	57.69%	60.51%
	2018	Outer regional & remote	59.07%	2.67%	53.83%	64.31%
	2019	Metro & inner regional	59.18%	0.79%	57.63%	60.74%
	2019	Outer regional & remote	65.79%	2.95%	60.01%	71.57%
	2020	Metro & inner regional	59.82%	0.88%	58.09%	61.54%
	2020	Outer regional & remote	58.26%	3.47%	51.46%	65.06%
	2021	Metro & inner regional	58.92%	0.73%	57.48%	60.35%
	2021	Outer regional & remote	64.79%	2.54%	59.82%	69.76%
	2022	Metro & inner regional	62.01%	0.69%	60.66%	63.35%
	2022	Outer regional & remote	69.39%	2.24%	65.00%	73.77%
<150 mins of moderate/vigorous activity p/w	2017	Metro & inner regional	33.88%	0.66%	32.59%	35.18%
	2017	Outer regional & remote	40.88%	2.83%	35.32%	46.44%
	2018	Metro & inner regional	32.16%	0.68%	30.82%	33.50%
	2018	Outer regional & remote	39.23%	2.58%	34.19%	44.28%
	2019	Metro & inner regional	31.42%	0.76%	29.94%	32.90%
	2019	Outer regional & remote	34.33%	2.76%	28.92%	39.73%
	2020	Metro & inner regional	31.23%	0.82%	29.62%	32.83%
	2020	Outer regional & remote	35.84%	3.18%	29.61%	42.06%
	2021	Metro & inner regional	30.00%	0.71%	28.62%	31.39%
	2021	Outer regional & remote	37.54%	2.56%	32.51%	42.56%
	2022	Metro & inner regional	32.51%	0.66%	31.22%	33.80%
	2022	Outer regional & remote	36.43%	2.47%	31.58%	41.28%
150 -300 mins of moderate/vigorous activity p/w	2017	Metro & inner regional	16.43%	0.55%	15.36%	17.50%

	2017	Outer regional & remote	15.51%	2.21%	11.18%	19.84%
	2018	Metro & inner regional	17.78%	0.59%	16.63%	18.93%
	2018	Outer regional & remote	17.16%	2.13%	12.99%	21.32%
	2019	Metro & inner regional	16.17%	0.62%	14.96%	17.38%
	2019	Outer regional & remote	17.20%	2.57%	12.15%	22.24%
	2020	Metro & inner regional	17.32%	0.71%	15.93%	18.70%
	2020	Outer regional & remote	14.83%	2.30%	10.32%	19.34%
	2021	Metro & inner regional	16.55%	0.56%	15.45%	17.64%
	2021	Outer regional & remote	14.44%	1.85%	10.81%	18.08%
	2022	Metro & inner regional	16.45%	0.54%	15.40%	17.50%
	2022	Outer regional & remote	16.04%	1.99%	12.13%	19.94%
>300 mins of moderate/vigorous activity p/w	2017	Metro & inner regional	49.69%	0.70%	48.30%	51.07%
	2017	Outer regional & remote	43.61%	2.88%	37.97%	49.24%
	2018	Metro & inner regional	50.06%	0.73%	48.63%	51.49%
	2018	Outer regional & remote	43.61%	2.71%	38.30%	48.92%
	2019	Metro & inner regional	52.41%	0.80%	50.84%	53.99%
	2019	Outer regional & remote	48.48%	3.15%	42.31%	54.65%
	2020	Metro & inner regional	51.46%	0.91%	49.67%	53.24%
	2020	Outer regional & remote	49.33%	3.45%	42.57%	56.10%
	2021	Metro & inner regional	53.45%	0.75%	51.98%	54.92%
	2021	Outer regional & remote	48.02%	2.62%	42.89%	53.15%
	2022	Metro & inner regional	51.05%	0.70%	49.66%	52.43%
	2022	Outer regional & remote	47.53%	2.52%	42.58%	52.48%
K10 scores 22+	2017	Metro & inner regional	15.22%	0.54%	14.16%	16.27%
	2017	Outer regional & remote	16.12%	2.30%	11.62%	20.62%
	2019	Metro & inner regional	17.92%	0.65%	16.64%	19.21%
	2019	Outer regional & remote	16.61%	2.61%	11.48%	21.73%
	2020	Metro & inner regional	16.99%	0.71%	15.60%	18.37%
	2020	Outer regional & remote	13.53%	2.42%	8.80%	18.27%
	2021	Metro & inner regional	16.84%	0.59%	15.69%	17.98%
	2021	Outer regional & remote	17.01%	2.06%	12.96%	21.05%
Poor/very poor health	2017	Metro & inner regional	7.78%	0.36%	7.07%	8.48%
	2017	Outer regional & remote	8.12%	1.48%	5.22%	11.02%
	2018	Metro & inner regional	7.89%	0.37%	7.16%	8.61%
	2018	Outer regional & remote	10.06%	1.61%	6.91%	13.21%
	2019	Metro & inner regional	7.59%	0.41%	6.79%	8.40%
	2019	Outer regional & remote	7.72%	1.29%	5.19%	10.25%
	2020	Metro & inner regional	5.20%	0.35%	4.51%	5.89%
	2020	Outer regional & remote	5.79%	1.10%	3.62%	7.95%
	2021	Metro & inner regional	5.13%	0.31%	4.53%	5.74%
	2021	Outer regional & remote	5.93%	1.16%	3.64%	8.21%
	2022	Metro & inner regional	8.74%	0.38%	7.98%	9.49%
	2022	Outer regional & remote	9.43%	1.36%	6.77%	12.09%

Supplementary Table 7

Table S7. Weighted prevalence of health risk behaviours, BMI category, and wellbeing indicators in NSW adults by Education, by year, 2017-2022

Outcome	Year	Education category	Prevalence (%)	Standard Error	95% CI (lower)	95%CI (upper)
Alcohol (11+ serves of alcohol p/w)	2017	High school year 10	12.48%	0.91%	10.69%	14.27%
	2017	High school year 12/TAFE/Diploma	15.45%	0.72%	14.04%	16.86%
	2017	University	12.72%	0.76%	11.22%	14.21%
	2018	High school year 10	11.51%	0.91%	9.73%	13.29%
	2018	High school year 12/TAFE/Diploma	14.99%	0.73%	13.55%	16.43%
	2018	University	15.33%	0.85%	13.66%	16.99%
	2019	High school year 10	11.72%	1.01%	9.74%	13.70%
	2019	High school year 12/TAFE/Diploma	14.66%	0.73%	13.22%	16.09%
	2019	University	13.37%	0.89%	11.62%	15.12%
	2020	High school year 10	12.65%	1.58%	9.54%	15.75%
	2020	High school year 12/TAFE/Diploma	15.75%	0.92%	13.95%	17.55%
	2020	University	15.10%	1.00%	13.13%	17.07%
	2021	High school year 10	13.43%	1.17%	11.14%	15.72%
	2021	High school year 12/TAFE/Diploma	15.65%	0.89%	13.91%	17.39%
	2021	University	13.70%	0.66%	12.41%	15.00%
	2022	High school year 10	10.90%	1.09%	8.76%	13.05%
	2022	High school year 12/TAFE/Diploma	14.65%	0.68%	13.32%	15.98%
	2022	University	13.03%	0.66%	11.74%	14.32%
Current smoker	2017	High school year 10	22.87%	1.49%	19.95%	25.79%
	2017	High school year 12/TAFE/Diploma	18.92%	0.88%	17.19%	20.64%
	2017	University	8.13%	0.64%	6.87%	9.39%
	2018	High school year 10	22.87%	1.50%	19.93%	25.81%
	2018	High school year 12/TAFE/Diploma	16.76%	0.85%	15.08%	18.43%
	2018	University	9.74%	0.69%	8.38%	11.10%
	2019	High school year 10	23.56%	1.73%	20.17%	26.94%
	2019	High school year 12/TAFE/Diploma	18.64%	0.98%	16.72%	20.56%
	2019	University	9.28%	0.77%	7.78%	10.78%
	2020	High school year 10	20.90%	1.94%	17.10%	24.70%
	2020	High school year 12/TAFE/Diploma	16.99%	1.06%	14.91%	19.07%
	2020	University	7.50%	0.72%	6.08%	8.91%
	2021	High school year 10	23.20%	1.75%	19.76%	26.63%
	2021	High school year 12/TAFE/Diploma	14.00%	0.71%	12.60%	15.39%
	2021	University	7.22%	0.53%	6.18%	8.27%
	2022	High school year 10	20.98%	1.69%	17.66%	24.30%
	2022	High school year 12/TAFE/Diploma	13.46%	0.69%	12.12%	14.81%
	2022	University	6.89%	0.50%	5.90%	7.88%
Current eCigarette user	2017	High school year 10	1.33%	0.39%	0.57%	2.09%
	2017	High school year 12/TAFE/Diploma	1.17%	0.23%	0.71%	1.63%

	2017	University	0.72%	0.20%	0.34%	1.11%
	2018	High school year 10	1.07%	0.40%	0.28%	1.86%
	2018	High school year 12/TAFE/Diploma	1.77%	0.30%	1.18%	2.36%
	2018	University	1.31%	0.29%	0.75%	1.87%
	2019	High school year 10	2.21%	0.67%	0.90%	3.52%
	2019	High school year 12/TAFE/Diploma	2.25%	0.38%	1.50%	2.99%
	2019	University	1.49%	0.33%	0.83%	2.14%
	2020	High school year 10	1.92%	0.69%	0.56%	3.27%
	2020	High school year 12/TAFE/Diploma	2.97%	0.49%	2.00%	3.93%
	2020	University	1.15%	0.28%	0.60%	1.70%
	2021	High school year 10	7.06%	1.18%	4.75%	9.37%
	2021	High school year 12/TAFE/Diploma	6.39%	0.56%	5.28%	7.49%
	2021	University	3.67%	0.46%	2.78%	4.57%
	2022	High school year 10	8.02%	1.23%	5.60%	10.44%
	2022	High school year 12/TAFE/Diploma	7.41%	0.54%	6.35%	8.48%
	2022	University	4.90%	0.46%	4.00%	5.80%
BMI (healthy weight)	2017	High school year 10	38.93%	1.57%	35.84%	42.02%
	2017	High school year 12/TAFE/Diploma	40.31%	1.05%	38.26%	42.37%
	2017	University	47.95%	1.14%	45.72%	50.17%
	2018	High school year 10	40.14%	1.61%	36.99%	43.30%
	2018	High school year 12/TAFE/Diploma	38.66%	1.06%	36.57%	40.74%
	2018	University	47.95%	1.15%	45.70%	50.20%
	2019	High school year 10	38.59%	1.86%	34.94%	42.23%
	2019	High school year 12/TAFE/Diploma	39.11%	1.17%	36.82%	41.40%
	2019	University	45.82%	1.27%	43.34%	48.31%
	2020	High school year 10	37.36%	2.24%	32.98%	41.75%
	2020	High school year 12/TAFE/Diploma	36.70%	1.29%	34.16%	39.23%
	2020	University	44.98%	1.34%	42.35%	47.60%
	2021	High school year 10	37.60%	1.99%	33.69%	41.50%
	2021	High school year 12/TAFE/Diploma	36.61%	1.12%	34.43%	38.80%
	2021	University	43.76%	1.00%	41.80%	45.72%
	2022	High school year 10	37.26%	2.08%	33.18%	41.35%
	2022	High school year 12/TAFE/Diploma	34.06%	1.01%	32.08%	36.04%
	2022	University	44.30%	0.98%	42.39%	46.21%
BMI (underweight)	2017	High school year 10	4.13%	0.63%	2.90%	5.36%
	2017	High school year 12/TAFE/Diploma	2.99%	0.39%	2.22%	3.76%
	2017	University	2.53%	0.37%	1.80%	3.26%
	2018	High school year 10	4.77%	0.82%	3.17%	6.38%
	2018	High school year 12/TAFE/Diploma	2.40%	0.33%	1.76%	3.04%
	2018	University	2.16%	0.34%	1.49%	2.83%
	2019	High school year 10	4.53%	0.83%	2.90%	6.16%
	2019	High school year 12/TAFE/Diploma	2.57%	0.38%	1.82%	3.33%
	2019	University	2.88%	0.44%	2.03%	3.74%
	2020	High school year 10	4.12%	1.11%	1.94%	6.29%
	2020	High school year 12/TAFE/Diploma	2.89%	0.45%	2.01%	3.77%
	2020	University	2.75%	0.48%	1.81%	3.68%

	2021	High school year 10	2.75%	0.69%	1.41%	4.10%
	2021	High school year 12/TAFE/Diploma	2.44%	0.45%	1.56%	3.33%
	2021	University	1.45%	0.23%	1.00%	1.90%
	2022	High school year 10	3.88%	0.84%	2.24%	5.52%
	2022	High school year 12/TAFE/Diploma	2.77%	0.41%	1.97%	3.57%
	2022	University	2.64%	0.32%	2.02%	3.26%
BMI (overweight)	2017	High school year 10	30.42%	1.42%	27.64%	33.19%
	2017	High school year 12/TAFE/Diploma	33.36%	1.00%	31.41%	35.31%
	2017	University	33.67%	1.09%	31.53%	35.82%
	2018	High school year 10	29.82%	1.42%	27.04%	32.60%
	2018	High school year 12/TAFE/Diploma	34.03%	1.04%	32.00%	36.06%
	2018	University	33.77%	1.09%	31.63%	35.90%
	2019	High school year 10	30.73%	1.67%	27.46%	34.01%
	2019	High school year 12/TAFE/Diploma	33.05%	1.12%	30.85%	35.24%
	2019	University	33.45%	1.21%	31.08%	35.81%
	2020	High school year 10	31.98%	1.98%	28.10%	35.87%
	2020	High school year 12/TAFE/Diploma	33.96%	1.23%	31.54%	36.38%
	2020	University	35.29%	1.29%	32.76%	37.83%
	2021	High school year 10	33.08%	1.92%	29.31%	36.85%
	2021	High school year 12/TAFE/Diploma	34.56%	1.01%	32.58%	36.53%
	2021	University	35.62%	0.97%	33.71%	37.52%
	2022	High school year 10	32.11%	1.91%	28.36%	35.85%
	2022	High school year 12/TAFE/Diploma	34.14%	0.99%	32.19%	36.08%
	2022	University	34.39%	0.94%	32.55%	36.24%
BMI (Obese)	2017	High school year 10	26.52%	1.36%	23.86%	29.18%
	2017	High school year 12/TAFE/Diploma	23.33%	0.87%	21.63%	25.03%
	2017	University	15.85%	0.85%	14.18%	17.51%
	2018	High school year 10	25.26%	1.27%	22.77%	27.76%
	2018	High school year 12/TAFE/Diploma	24.91%	0.94%	23.08%	26.75%
	2018	University	16.13%	0.83%	14.49%	17.76%
	2019	High school year 10	26.15%	1.53%	23.16%	29.14%
	2019	High school year 12/TAFE/Diploma	25.27%	1.02%	23.28%	27.27%
	2019	University	17.85%	0.99%	15.91%	19.78%
	2020	High school year 10	26.53%	1.74%	23.11%	29.95%
	2020	High school year 12/TAFE/Diploma	26.46%	1.17%	24.17%	28.74%
	2020	University	16.98%	1.01%	15.00%	18.97%
	2021	High school year 10	26.57%	1.71%	23.21%	29.93%
	2021	High school year 12/TAFE/Diploma	26.39%	0.92%	24.59%	28.18%
	2021	University	19.17%	0.78%	17.65%	20.70%
	2022	High school year 10	26.75%	1.79%	23.24%	30.27%
	2022	High school year 12/TAFE/Diploma	29.03%	0.94%	27.18%	30.88%
	2022	University	18.67%	0.79%	17.13%	20.22%
<3 serves of vegetables p/d	2017	High school year 10	74.05%	1.25%	71.60%	76.50%
	2017	High school year 12/TAFE/Diploma	67.00%	0.97%	65.10%	68.90%

	2017	University	57.60%	1.11%	55.42%	59.79%
	2018	High school year 10	78.41%	1.09%	76.27%	80.56%
	2018	High school year 12/TAFE/Diploma	69.16%	0.97%	67.25%	71.07%
	2018	University	57.61%	1.14%	55.37%	59.85%
	2019	High school year 10	76.01%	1.41%	73.24%	78.77%
	2019	High school year 12/TAFE/Diploma	71.10%	1.05%	69.04%	73.17%
	2019	University	58.67%	1.24%	56.23%	61.10%
	2020	High school year 10	78.17%	1.55%	75.13%	81.21%
	2020	High school year 12/TAFE/Diploma	71.18%	1.17%	68.88%	73.48%
	2020	University	61.75%	1.31%	59.18%	64.31%
	2021	High school year 10	78.42%	1.61%	75.27%	81.58%
	2021	High school year 12/TAFE/Diploma	73.80%	0.95%	71.94%	75.66%
	2021	University	61.62%	0.97%	59.72%	63.53%
	2022	High school year 10	79.16%	1.83%	75.57%	82.76%
	2022	High school year 12/TAFE/Diploma	76.16%	0.87%	74.46%	77.86%
	2022	University	64.39%	0.94%	62.55%	66.23%
<2 serves of fruit p/d	2017	High school year 10	59.05%	1.49%	56.13%	61.98%
	2017	High school year 12/TAFE/Diploma	55.01%	1.07%	52.92%	57.09%
	2017	University	49.69%	1.15%	47.43%	51.95%
	2018	High school year 10	63.63%	1.45%	60.78%	66.47%
	2018	High school year 12/TAFE/Diploma	61.78%	1.05%	59.73%	63.84%
	2018	University	54.37%	1.16%	52.09%	56.66%
	2019	High school year 10	65.23%	1.66%	61.98%	68.48%
	2019	High school year 12/TAFE/Diploma	61.38%	1.16%	59.11%	63.66%
	2019	University	55.36%	1.28%	52.86%	57.86%
	2020	High school year 10	62.34%	2.02%	58.37%	66.30%
	2020	High school year 12/TAFE/Diploma	61.64%	1.30%	59.09%	64.19%
	2020	University	56.92%	1.36%	54.26%	59.58%
	2021	High school year 10	64.03%	1.95%	60.21%	67.85%
	2021	High school year 12/TAFE/Diploma	61.13%	1.16%	58.87%	63.40%
	2021	University	56.09%	1.00%	54.13%	58.06%
	2022	High school year 10	68.97%	1.95%	65.15%	72.79%
	2022	High school year 12/TAFE/Diploma	64.38%	1.02%	62.38%	66.38%
	2022	University	58.66%	0.98%	56.75%	60.57%
<150 mins of moderate/vigorous activity p/w	2017	High school year 10	44.18%	1.51%	41.21%	47.14%
	2017	High school year 12/TAFE/Diploma	36.28%	1.01%	34.31%	38.26%
	2017	University	27.63%	1.00%	25.66%	29.59%
	2018	High school year 10	43.55%	1.53%	40.56%	46.54%
	2018	High school year 12/TAFE/Diploma	36.35%	1.08%	34.23%	38.47%
	2018	University	24.13%	0.96%	22.24%	26.02%
	2019	High school year 10	43.11%	1.83%	39.53%	46.69%
	2019	High school year 12/TAFE/Diploma	34.77%	1.09%	32.63%	36.91%
	2019	University	24.00%	1.10%	21.84%	26.15%
	2020	High school year 10	43.40%	2.04%	39.39%	47.40%

	2020	High school year 12/TAFE/Diploma	35.11%	1.27%	32.62%	37.59%
	2020	University	23.89%	1.18%	21.58%	26.19%
	2021	High school year 10	41.91%	2.02%	37.96%	45.86%
	2021	High school year 12/TAFE/Diploma	34.25%	1.17%	31.96%	36.54%
	2021	University	23.89%	0.86%	22.20%	25.58%
	2022	High school year 10	43.73%	2.12%	39.57%	47.89%
	2022	High school year 12/TAFE/Diploma	36.00%	1.03%	33.99%	38.02%
	2022	University	26.82%	0.86%	25.15%	28.50%
150 -300 mins of moderate/vigorous activity p/w	2017	High school year 10	15.18%	1.12%	12.99%	17.38%
	2017	High school year 12/TAFE/Diploma	17.09%	0.85%	15.43%	18.75%
	2017	University	16.22%	0.83%	14.60%	17.85%
	2018	High school year 10	16.23%	1.25%	13.77%	18.69%
	2018	High school year 12/TAFE/Diploma	17.13%	0.86%	15.45%	18.81%
	2018	University	18.99%	0.92%	17.19%	20.79%
	2019	High school year 10	15.51%	1.35%	12.87%	18.15%
	2019	High school year 12/TAFE/Diploma	15.96%	0.92%	14.16%	17.76%
	2019	University	16.75%	0.95%	14.89%	18.62%
	2020	High school year 10	14.41%	1.46%	11.55%	17.28%
	2020	High school year 12/TAFE/Diploma	17.57%	1.07%	15.48%	19.66%
	2020	University	17.74%	1.07%	15.64%	19.84%
	2021	High school year 10	14.39%	1.61%	11.23%	17.55%
	2021	High school year 12/TAFE/Diploma	16.29%	0.85%	14.63%	17.96%
	2021	University	17.05%	0.79%	15.51%	18.60%
	2022	High school year 10	16.46%	1.60%	13.32%	19.60%
	2022	High school year 12/TAFE/Diploma	15.45%	0.81%	13.87%	17.03%
	2022	University	17.25%	0.77%	15.74%	18.76%
>300 mins of moderate/vigorous activity p/w	2017	High school year 10	40.64%	1.53%	37.64%	43.63%
	2017	High school year 12/TAFE/Diploma	46.63%	1.05%	44.57%	48.69%
	2017	University	56.15%	1.10%	53.99%	58.32%
	2018	High school year 10	40.23%	1.63%	37.03%	43.42%
	2018	High school year 12/TAFE/Diploma	46.52%	1.07%	44.42%	48.63%
	2018	University	56.88%	1.12%	54.68%	59.09%
	2019	High school year 10	41.38%	1.87%	37.71%	45.05%
	2019	High school year 12/TAFE/Diploma	49.27%	1.16%	47.00%	51.53%
	2019	University	59.25%	1.24%	56.82%	61.68%
	2020	High school year 10	42.19%	2.16%	37.95%	46.43%
	2020	High school year 12/TAFE/Diploma	47.33%	1.34%	44.70%	49.96%
	2020	University	58.38%	1.32%	55.78%	60.97%
	2021	High school year 10	43.70%	2.05%	39.68%	47.71%
	2021	High school year 12/TAFE/Diploma	49.45%	1.17%	47.16%	51.75%
	2021	University	59.06%	0.99%	57.12%	61.00%
	2022	High school year 10	39.82%	2.04%	35.81%	43.82%
	2022	High school year 12/TAFE/Diploma	48.55%	1.06%	46.46%	50.63%

	2022	University	55.93%	0.97%	54.03%	57.82%
K10 scores 22+	2017	High school year 10	24.28%	1.49%	21.35%	27.21%
	2017	High school year 12/TAFE/Diploma	17.06%	0.85%	15.40%	18.72%
	2017	University	9.93%	0.69%	8.58%	11.28%
	2019	High school year 10	26.69%	1.74%	23.27%	30.10%
	2019	High school year 12/TAFE/Diploma	20.98%	1.03%	18.97%	23.00%
	2019	University	11.66%	0.87%	9.96%	13.36%
	2020	High school year 10	22.93%	1.97%	19.07%	26.80%
	2020	High school year 12/TAFE/Diploma	19.04%	1.09%	16.91%	21.17%
	2020	University	12.72%	0.95%	10.85%	14.59%
	2021	High school year 10	23.01%	1.73%	19.62%	26.40%
	2021	High school year 12/TAFE/Diploma	20.65%	0.99%	18.71%	22.59%
	2021	University	11.85%	0.65%	10.57%	13.12%
Poor/very poor health	2017	High school year 10	10.70%	0.85%	9.04%	12.37%
	2017	High school year 12/TAFE/Diploma	8.46%	0.56%	7.36%	9.55%
	2017	University	5.53%	0.53%	4.50%	6.56%
	2018	High school year 10	11.53%	0.91%	9.75%	13.32%
	2018	High school year 12/TAFE/Diploma	9.33%	0.61%	8.13%	10.53%
	2018	University	4.89%	0.49%	3.94%	5.85%
	2019	High school year 10	12.46%	1.19%	10.14%	14.79%
	2019	High school year 12/TAFE/Diploma	8.60%	0.63%	7.37%	9.84%
	2019	University	4.43%	0.50%	3.44%	5.42%
	2020	High school year 10	7.44%	0.97%	5.54%	9.34%
	2020	High school year 12/TAFE/Diploma	6.25%	0.57%	5.13%	7.37%
	2020	University	3.31%	0.44%	2.45%	4.17%
	2021	High school year 10	8.04%	1.04%	6.01%	10.08%
	2021	High school year 12/TAFE/Diploma	6.24%	0.53%	5.21%	7.27%
	2021	University	3.30%	0.35%	2.61%	3.99%
	2022	High school year 10	11.43%	1.14%	9.21%	13.66%
	2022	High school year 12/TAFE/Diploma	9.69%	0.60%	8.52%	10.87%
	2022	University	7.11%	0.52%	6.09%	8.14%

Supplementary Figure 1

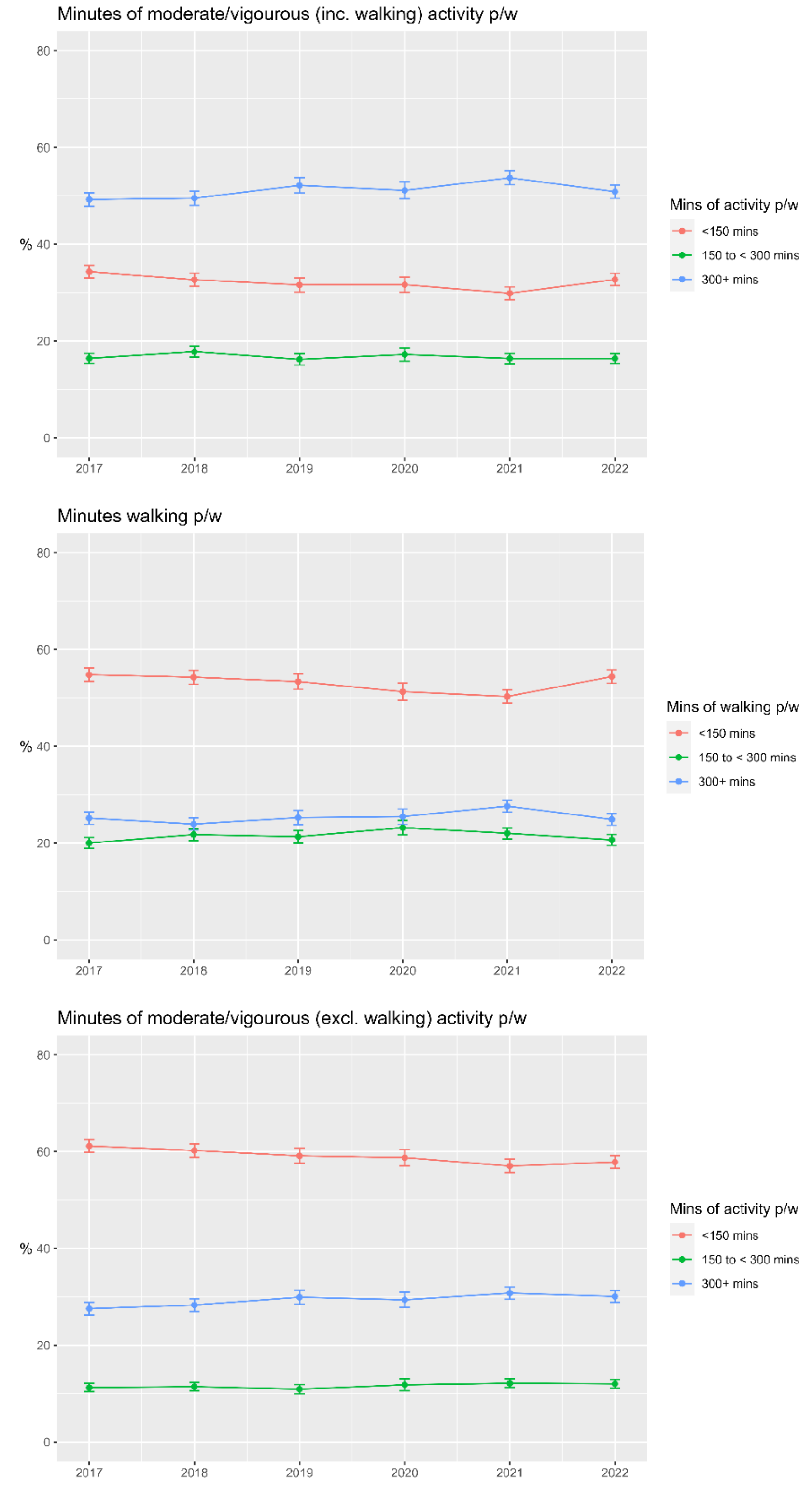

Figure S1. Supplementary analysis of physical activity (MVPA) with and without walking (weighted, unadjusted prevalence)

Supplementary Figure 2

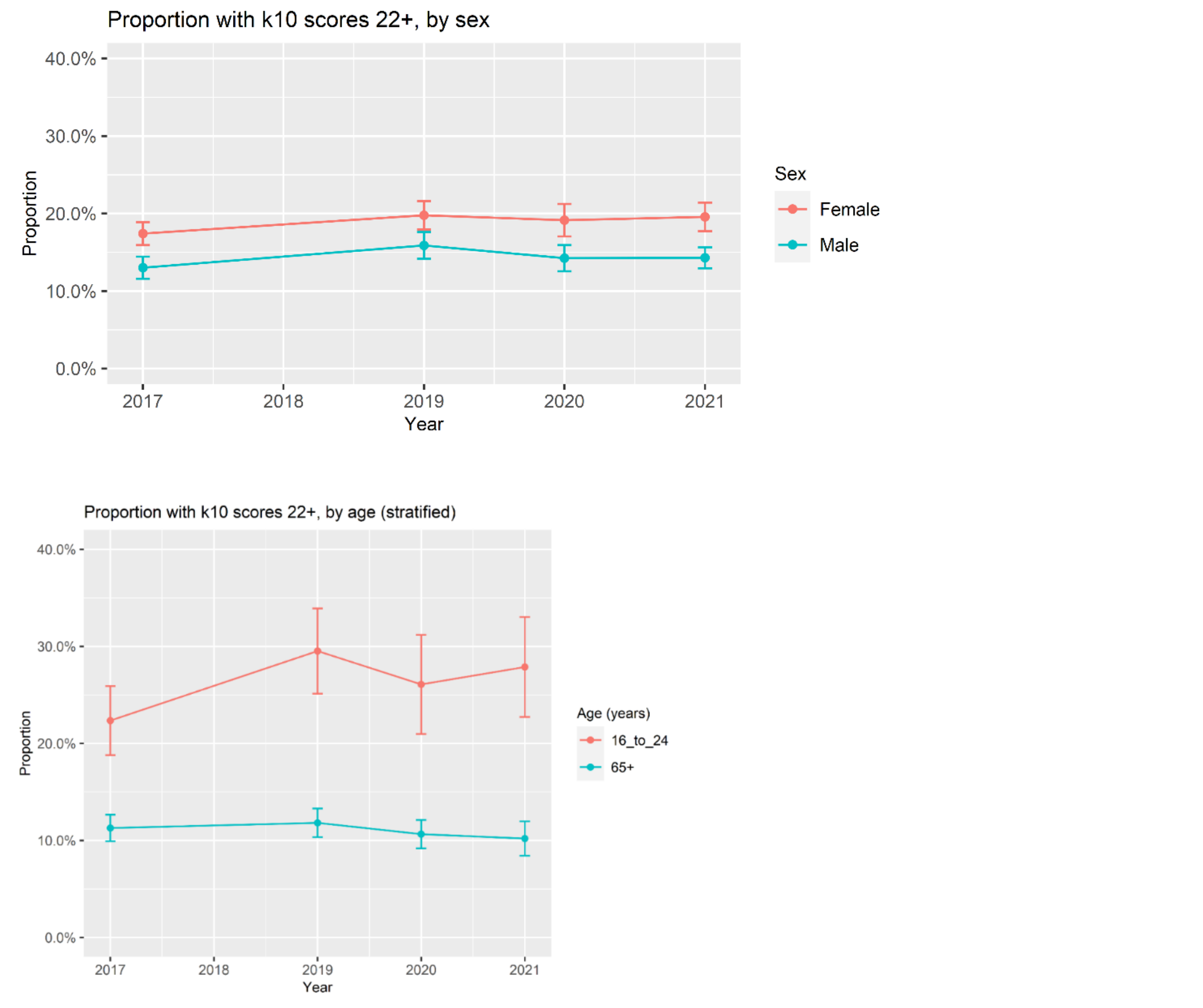

Figure S2. Supplementary analyses of high/very high psychological distress (K10 ≥22) according to sex and age group (weighted, unadjusted prevalence).

Supplementary Figure 3

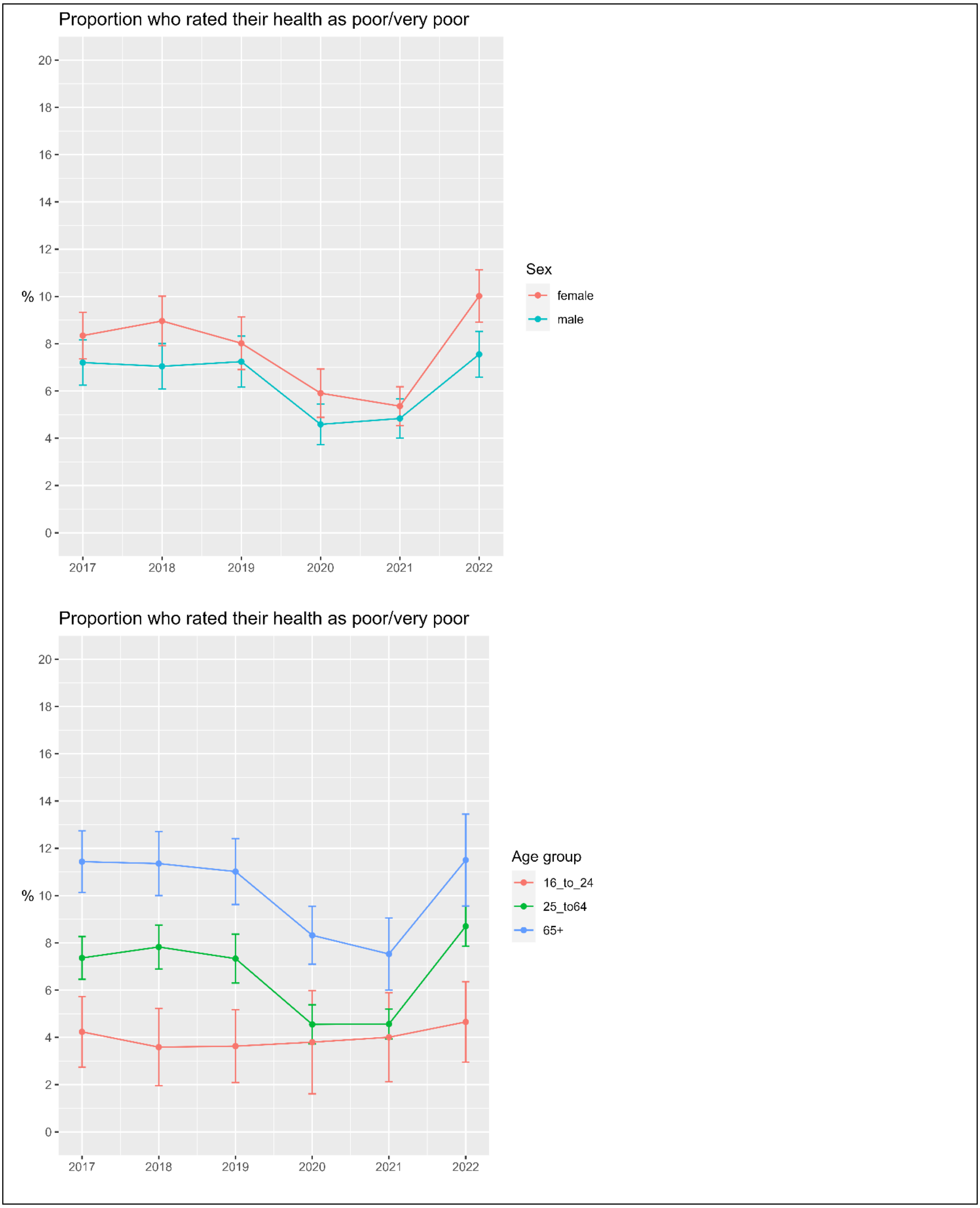

Figure S3. Supplementary analyses of poor/very poor self-rated health according to sex and age group (weighted, unadjusted prevalence).

Supplementary Figure 4

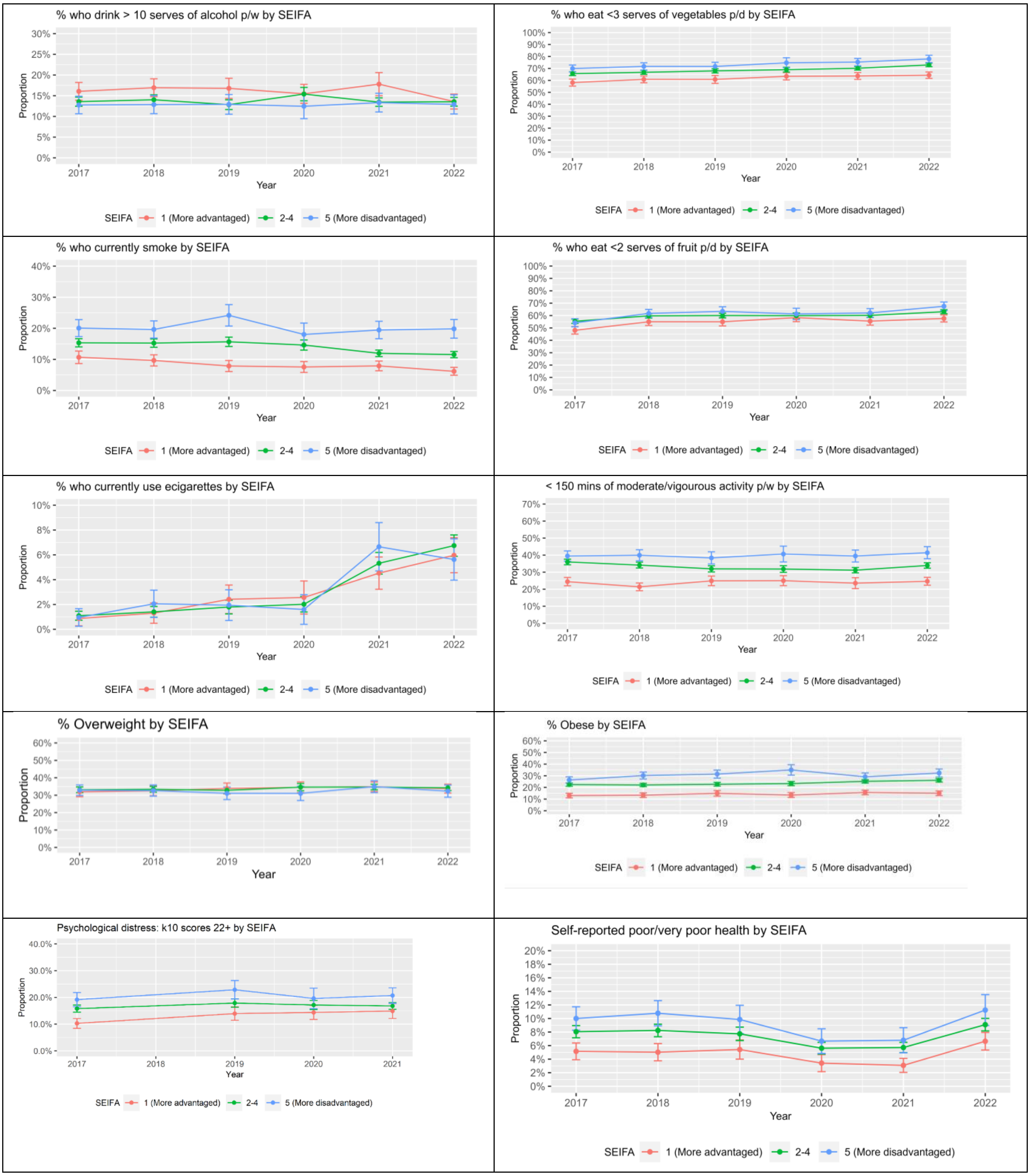

Figure S4. Weighted prevalence* of health behaviours, overweight, obesity, and wellbeing indicators according to relative socio-economic disadvantage (SEIFA IRSD), in NSW adults by year, 2017-2022

*Adjusted for age, sex, language spoken, and country of birth based on Human Development Index.

Supplementary Figure 5

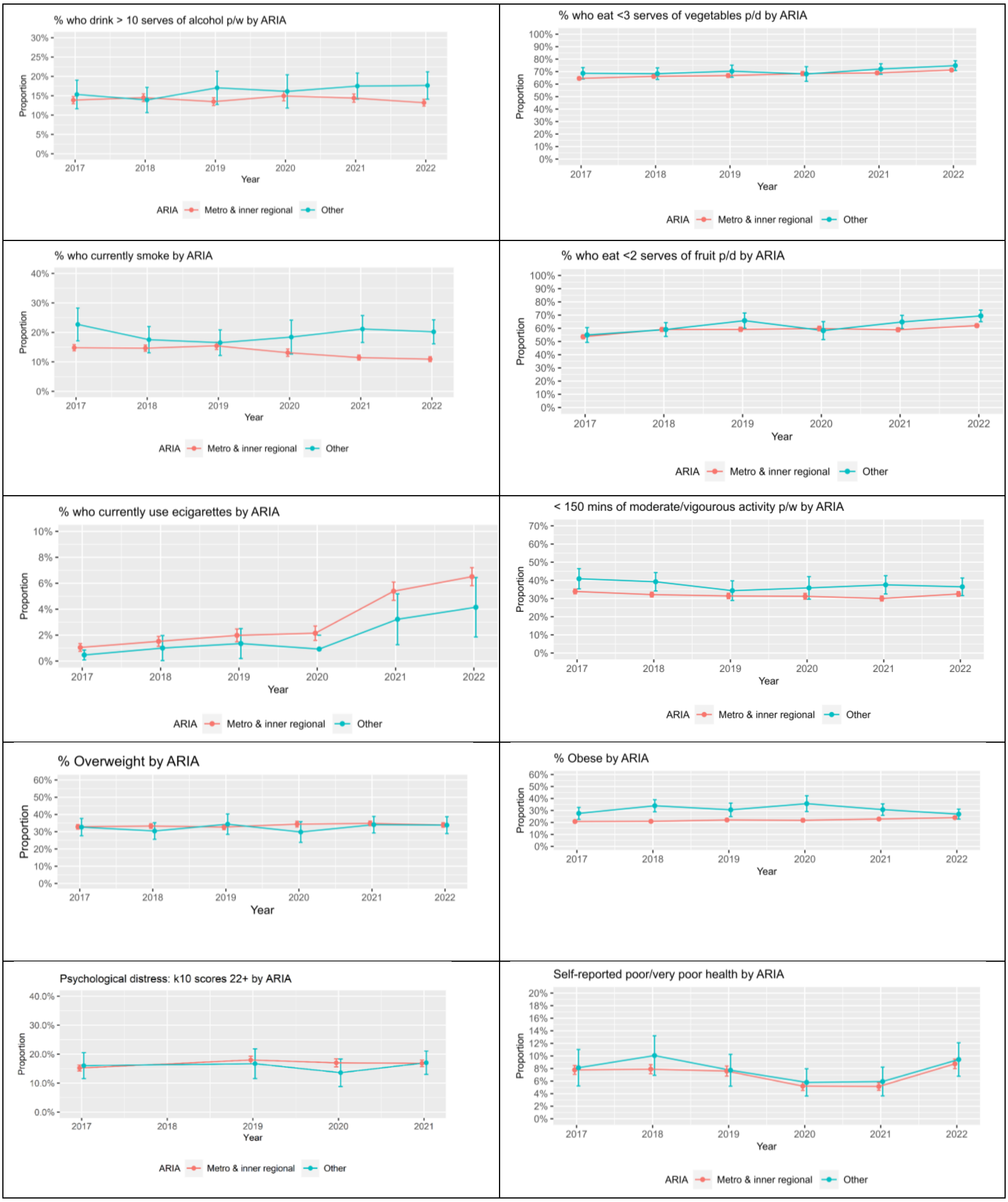

Figure S5. Weighted prevalence* of health behaviours, overweight, obesity, and wellbeing indicators according to geographical remoteness (ARIA), in NSW adults by year, 2017-2022

*Adjusted for age, sex, language spoken, and country of birth based on Human Development Index.

Supplementary Figure 6

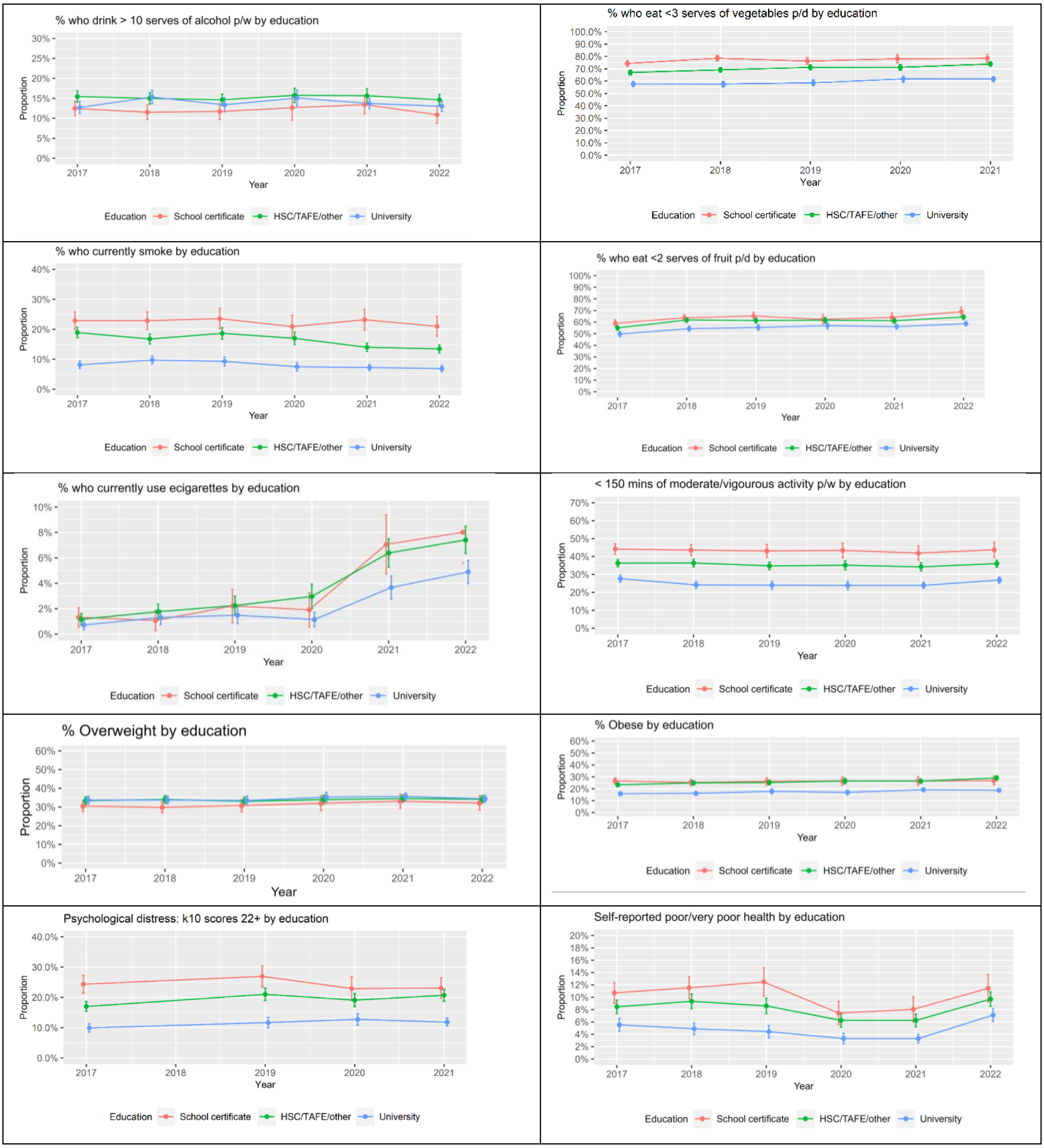

Figure S6. Weighted prevalence* of health behaviours, overweight, obesity, and wellbeing indicators according to education attainment, in NSW adults by year, 2017-2022

*Adjusted for age, sex, language spoken, and country of birth based on Human Development Index.
